# Inhibition of carnitine palmitoyl-transferase 1 is a potential target in a mouse model of Parkinson’s disease

**DOI:** 10.1038/s41531-023-00450-y

**Published:** 2023-01-21

**Authors:** Michael Sloth Trabjerg, Dennis Christian Andersen, Pam Huntjens, Kasper Mørk, Nikolaj Warming, Ulla Bismark Kullab, Marie-Louise Nibelius Skjønnemand, Michal Krystian Oklinski, Kirsten Egelund Oklinski, Luise Bolther, Lona J. Kroese, Colin E. J. Pritchard, Ivo J. Huijbers, Angelique Corthals, Mads Toft Søndergaard, Henrik Bech Kjeldal, Cecilie Fjord Morre Pedersen, John Dirk Vestergaard Nieland

**Affiliations:** 1 Laboratory of Molecular Pharmacology, Department of Health Science and Technology, Aalborg University, Aalborg, Denmark; 2 Mouse Clinic for Cancer and Aging (MCCA) Transgenic Facility, The Netherlands Cancer Institute, 1066 CX Amsterdam, The Netherlands; 3 Department of Science, John Jay College of Criminal Justice, City University of New York, New York, NY 10019 USA; 4 DNASense, Aalborg, Denmark

**Keywords:** Parkinson's disease, Molecular biology, Drug discovery, Neuroscience, Pathogenesis

## Abstract

Glucose metabolism is dysregulated in Parkinson’s disease (PD) causing a shift toward the metabolism of lipids. Carnitine palmitoyl-transferase 1A (CPT1A) regulates the key step in the metabolism of long-chain fatty acids. The aim of this study is to evaluate the effect of downregulating CPT1, either genetically with a *Cpt1a* P479L mutation or medicinally on PD using chronic rotenone mouse models using C57Bl/6J and *Park2* knockout mice. We show that *Cpt1a* P479L mutant mice are resistant to rotenone-induced PD, and that inhibition of CPT1 is capable of restoring neurological function, normal glucose metabolism, and alleviate markers of PD in the midbrain. Furthermore, we show that downregulation of lipid metabolism via CPT1 alleviates pathological motor and non-motor behavior, oxidative stress, and disrupted glucose homeostasis in *Park2* knockout mice. Finally, we confirm that rotenone induces gut dysbiosis in C57Bl/6J and, for the first time, in *Park2* knockout mice. We show that this dysbiosis is alleviated by the downregulation of the lipid metabolism via CPT1.

## Introduction

Parkinson’s disease (PD) is the second most common neurodegenerative disorder characterized by the degeneration of dopaminergic (DA) neurons in substantia nigra pars compacta. The death of DA neurons results in characteristic motor symptoms such as tremor, rigidity, bradykinesia, and loss of muscle strength^1^. Non-motor symptoms such as cognitive dysfunction and depression often precede motor symptoms by more than a decade^1^. Mechanisms such as mitochondrial dysfunction, inflammation, autoantibodies, oxidative stress, protein aggregation, and hypoxia play a role in the establishment and progression of the disease^1–4^.

Metabolic dysfunction, however, might be a key mechanism in the development and progression of PD. Impaired glucose metabolism is indicated early in the disease, and this impairment is associated with an increase in microglial activation^5,6^. These findings are supported by an alteration in fatty acid metabolites observed in PD patients, indicating a modified β-oxidation^7–11^. Furthermore, rotenone, a pesticide used to induce PD in mice and rats, upregulates β-oxidation in neurons^12^ and systemically, including liver and muscles^13^. In accordance, the autosomal recessive *PARK2-*mutation form of PD is characterized by upregulation of β-oxidation, and downregulation of glucose metabolism^14,15^. The key molecule in the β-oxidation pathway is carnitine palmitoyl-transferase 1 (CPT1) which regulates the transport of medium long-chain fatty acids over the outer mitochondrial membrane^16^. Three isoforms of CPT1 are identified; CPT1A, CPT1B, and CPT1C. CPT1A is expressed in the majority of cells, CPT1B is primarily expressed in muscles, and CPT1C is expressed in the brain. Upregulated lipid metabolism is essential for the survival of immune cells, and it enhances inflammation^17,18^. Furthermore, the production of acetyl-CoA through upregulated β-oxidation results in negative feedback to pyruvate and thereby glucose metabolism leading to a vicious cycle disrupting the cell metabolic homeostasis^19^.

Various mechanisms could disrupt the metabolic balance between glucose and lipids such as dysregulation of the hypothalamic–pituitary–adrenal (HPA) axis^20,21^, insulin resistance^22^, infection^23^, hypoxia^3^, micro hemorrhage, gut dysbiosis, and thereby the gut-brain axis^24,25^. Therefore, we tend to assume that PD is a multisystem disease affecting the CNS, rather than a function of a unique pathway^26^.

We have demonstrated that the application of a CPT1 antagonist is effective in animal models of multiple sclerosis (EAE) and depression through mechanisms such as downregulation of inflammation, autoantibody production, restoration of the myelin sheath, diminished iron-load, and restoring glucose metabolism^27–29^. We have also previously shown that mice with homozygote CPT1A P479L mutation, resulting in 22% CPT1A activity^27^, are resistant to the induction of EAE^30^.

The overall aim of this study is to evaluate the effects of downregulated CPT1 and CPT1A lipid metabolism in multiple in vivo models mimicking PD by pharmacological and genetic methods. Furthermore, we aim to investigate the effect of downregulating CPT1 in a chronic rotenone and a *Park2* knockout mouse model without or combined with rotenone.

## Results

**Fig. 1:**
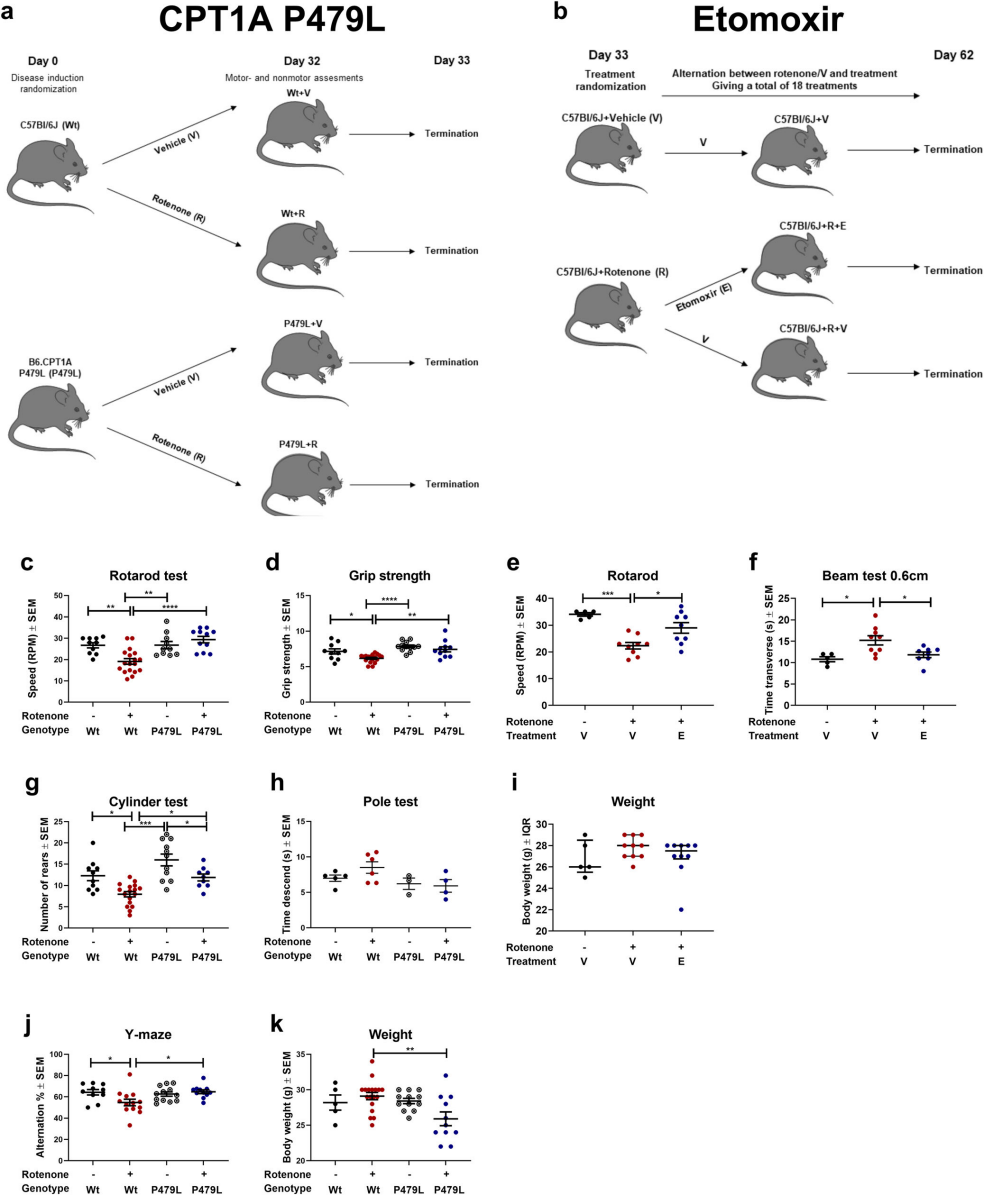
CPT1A P479L mice show resistance to chronic rotenone-induced motor and non-motor impairment and downregulation of CPT1 activity using etomoxir ameliorates rotenone-induced motor impairment. **a** Male C57Bl/6J and CPT1A P479L mice received vehicle (carboxymethylcellulose sodium salt, 0.5%) or rotenone (30 mg/kg) for 32 days and were then evaluated by motor and non-motor behavior tests. **b** Male C57Bl/6J mice received vehicle (carboxymethylcellulose sodium salt, 0.5%) or rotenone (30 m/kg) for 32 days followed by an alternating treatment regime with etomoxir/vehicle and rotenone until termination at day 62. **c** Mean rounds per minute on the rotarod test (*n* = 10–18). **d** Mean normalized grip strength (*n* = 10–18). **e** Mean rounds per minute on the rotarod test at day 60 (*n* = 5–9). **f** Mean time to transverse the 0.6 cm beam at day 42 (*n* = 5–9). **g** Mean number of rears in the cylinder test (*n* = 9–17). **h** Time to descend the pole in the pole test (*n* = 3-6). **i** Mean weight of mice at day 62 in grams (*n* = 5–9). **j** Visuospatial memory based on the Y-maze test measured as mean spontaneous alternation percentage (*n* = 10–13). **k** Mean weight of mice at day 32 in grams (*n* = 5–18). Significant differences for behavior; *p ≤ 0.05; **p ≤ 0.01; ***p ≤ 0.001; ****p ≤ 0.0001. Wt wildtype, P479L *Cpt1a* P479L homozygote genotype, E etomoxir, R rotenone, V Vehicle, RPM rounds per minute, SAP spontaneous alternation percentage. Center line = mean, and whiskers = standard error of the mean except for **i**, center line = median and whiskers = interquartile range. Statistics: two-way or one-way ANOVA followed by Tukey post hoc test or Kruskal–Wallis test followed by Dunn post hoc test. We acknowledge Servier Medical Art for the mouse illustration, with the following license: https://creativecommons.org/licenses/by/3.0/. No changes were made to the drawing.

To evaluate whether CPT1 and CPT1A lipid metabolism play a role in the induction and progression of PD-like disease we established, and combined a variety of mouse models mimicking some aspects of PD. We used the rotenone model as this induces multiple characteristics of PD, as described in the next paragraph. In the first study (Fig. 1a), we established a mouse model with a CPT1A P479L homozygote mutation to evaluate whether this affected the induction of PD-like disease. In the second study (Fig. 1b), we continuously exposed wildtype (Wt) mice to rotenone and tested the effect of downregulating CPT1 by a pharmacological blocker following the induction of PD-like characteristics. In the third study (Fig. 4a), we exposed Wt mice to rotenone for 32 days, terminated the exposure and assessed the effect of downregulating CPT1A by a pharmacological blocker to evaluate whether this was effective during washout of rotenone. In the fourth study (Fig. 6a), we used an autosomal recessive PD mouse model (PARK2 mutation), characterized by metabolic dysregulation (Fig. 5a), mimicking some aspects of familiar PD to evaluate whether downregulation of CPT1 by a pharmacological blocker had any effect on behavior and pathological hallmarks of PD. Finally, we exposed PARK2 mice to rotenone for 32 days (Fig. 7a) and evaluated whether pharmacological downregulation of CPT1 had any effect on behavior and pathological hallmarks (Fig. 8a). The motor and non-motor tests used to evaluate pathological behavior in the different models had some dissimilarities because not all the tests were established in the animal facility from the initiation of the first rotenone experiment (Fig. 1b), further information is available in the methods.

### CPT1A P479L mutant mice show resistance against chronic rotenone disease induction

In the last decade, a robust chronic toxic rotenone mouse model has been established which induces some motor and biochemical alternations mimicking PD^31^. In this study, the chronic rotenone mouse model was established in C57BL/6J mice by administrating 30 mg/kg rotenone by oral gavage daily for 32 days. The success of the rotenone mouse model was evaluated through validated motor, sensorimotor and cognitive proficiency tests.

To elucidate whether the CPT1A P479L mutation protected against PD, a new mouse model with a *Cpt1a* P479L mutation was developed as previously published^30^. CPT1A P479L mutant mice (P479L+R) and Wt C57Bl/6J mice (Wt+R) received 32 days of oral rotenone as previously published in a pilot study^32^. Furthermore, C57Bl/6J and CPT1A P479L mice received vehicle (Wt+V, P479L+V) (Fig. 1a). At day 32, pathological motor and sensorimotor behaviors were evaluated by rotarod, grip strength, cylinder and pole test as these motor tests have been validated in the chronic rotenone mouse model and are surrogate markers for some of the motor abnormalities seen in PD patients^1,31,33,34^. The P479L+R mice did not develop any pathological motor behavior characteristic to rotenone exposure. P479L+R mice had significantly higher mean speed at the rotarod (29.4 ± 1.5 standard error of the mean (SEM)) (Fig. 1c), increased mean normalized grip strength (7.4 ± 0.4 SEM) (Fig. 1d) and mean spontaneous activity in the cylinder test (11.9 ± 0.8) (Fig. 1g) compared to Wt+R mice (mean speed 19.15 ± 1.3, *p* = 0.0001, mean grip strength 6.2 ± 0.1, *p* = 0.0024, mean spontaneous activity 7.9 ± 0.6, *p* = 0.03). This indicates that CPT1A P479L conferred resistance toward rotenone-induced motor deficits and decreased muscle strength, compared to Wt+R mice; hence, the CPT1A P479L mutation appears to provide protection against PD symptoms.

In addition, PD is characterized by non-motor symptoms including cognitive impairment^35^. Based on this, we evaluated whether the CPT1A P479L affected the presence of non-motor impairment in the form of visuospatial memory recognition following rotenone exposure. P479L+R mice had a significantly higher mean spontaneous alternation percentage (64.8 ± 1.7 SEM) compared to Wt+R mice (54.7 ± 3.1 SEM, *p* = 0.02) (Fig. 1j). Hence, the downregulation of CPT1A, which entails decreased activity, appears to be accompanied by increased resistance to the development of cognitive impairment. Wt+R mice developed both motor and non-motor impairment behavior compared to Wt+V mice in all tests (Fig. 1c, d, g, j). However, P479L+R mice did not develop any disease behavior compared to P479L+V mice, except for the cylinder test (Fig. 1g), indicating resistance to rotenone-induced disease induction. Taken together this demonstrated that the toxic chronic rotenone mouse model was induced successfully, and that CPT1A P479L conferred some resistance against non-motor neuron symptoms.

### CPT1A P479L mutation decreases glucose concentrations and changes the lipoprotein profile

**Fig. 2:**
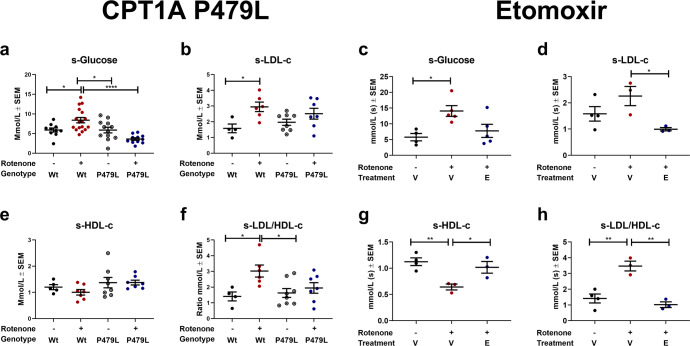
CPT1A P479L mutation and pharmacological CPT1 inhibition ameliorate rotenone-induced hyperglycemia and lipoprotein disturbances in mice. **a** Mean serum glucose levels measured in mmol/l (*n* = 10–17). **b** Mean serum LDL-c levels measured in mmol/l (*n* = 4–8). **c** Mean serum glucose levels measured in mmol/l (*n* = 4–5). **d** Mean serum LDL-c levels measured in mmol/l (*n* = 3–4). **e** Mean serum HDL-c levels measured in mmol/l (*n* = 5–8). **f** Mean ratio of serum LDL-c/HDL-c levels (*n* = 4–8). **g** Mean serum HDL-c levels measured in mmol/l (*n* = 3–4). **h** Mean ratio of serum LDL-c/HDL-c levels (*n* = 3–4). Significant differences for serum experiments: *p ≤ 0.05; **p ≤ 0.01. Wt wildtype, P479L *Cpt1a* P479L homozygote genotype, E etomoxir, R rotenone, V Vehicle. Center line = mean, and whiskers = standard error of the mean. Statistics: two-way ANOVA followed by Tukey post hoc test or one-way ANOVA followed by Tukey post hoc test.

Following the evaluation of pathological motor and non-motor behavior, we hypothesized that downregulation of CPT1 and specifically CPT1A would result in a shift toward glucose metabolism. Based on this hypothesis, we assessed serum levels of glucose in the different groups. P479L+R mice had significantly (*p* = 0.0001) lower mean glucose concentration (3.6 mmol/L ± 0.3 SEM) compared to Wt+R mice (8.4 mmol/L ± 0.7 SEM) (Fig. 2a). In addition, Wt+R mice had significantly (*p* = 0.035) higher mean glucose concentration (8.4 mmol/L ± 0.7 SEM) compared to Wt+V mice (5.9 mmol/L ± 0.5 SEM). This indicates that rotenone-induced hyperglycemia, which was attenuated by the P479L mutation. PD and neurodegenerative diseases are associated with disrupted homeostatic lipid composition^36^ and pathological interactions between lipoproteins and α-synuclein (α-syn) are implicated in PD^36^. In addition, increased oxidation of low-density lipoprotein (LDL) is associated with inflammation and oxidative stress^37^. Therefore, we evaluated the levels of LDL cholesterol (LDL-c) and high-density lipoprotein cholesterol (HDL-c) in serum from these mice. Wt+R mice had significantly (*p* = 0.03) higher mean LDL concentrations (3.0 mmol/L ± 0.3 SEM) compared to Wt+V mice (1.6 mmol/L ± 0.3 SEM) and significantly (*p* = 0.01) higher mean LDL/HDL ratio (3.0 ± 0.4 SEM) compared to both Wt+V (1.4 ± 0.3 SEM) and P479L+V mice (1.6 ± 0.3 SEM, *p* = 0.2) (Fig. 2b, e–f). This indicates that rotenone resulted in dysregulation of lipoprotein metabolism in Wt mice but not in P479L mice.

### CPT1A P479L mutation attenuates normalized gene expression and protein concentrations of PD biomarkers in the midbrain

**Fig. 3:**
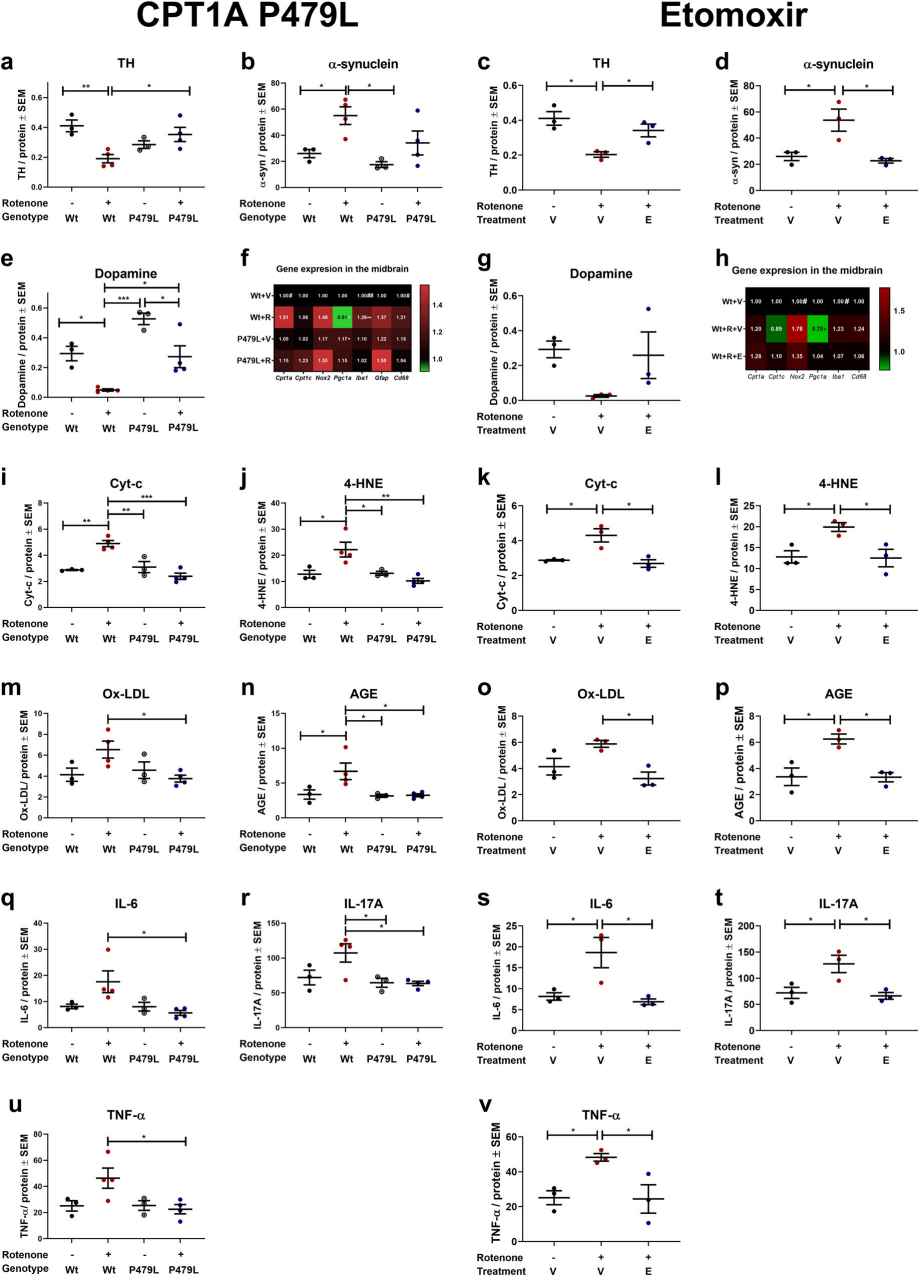
CPT1A P479L mutation and pharmacological CPT1 inhibition attenuate rotenone-induced pathological hallmarks of PD-like disease. **a** Mean TH levels in the midbrain measured in ng/mg total protein level (*n* = 3–4). **b** α-Synuclein levels in the midbrain expressed as pg/mg total protein level (*n* = 3–4). **c** TH levels in the midbrain expressed as mean ng/mg total protein (*n* = 3). **d** α-Synuclein levels in the midbrain expressed as mean pg/mg total protein (*n* = 3). **e** Dopamine levels in the midbrain expressed as ng/mg total protein (*n* = 3–4). **f** Heatmap illustrating mean fold gene expression change in the midbrain of *Cpt1a, Cpt1c, Nox2, Pgc1a, Iba1, Gfap* and *Cd68* (*n* = 4). **g** Dopamine levels in the midbrain expressed as mean ng/mg total protein (*n* = 3). **h** Heatmap illustrating mean fold gene expression change in the midbrain of *Cpt1a, Cpt1c, Nox2, Pgc1a, Iba1* and *Cd68* (*n* = 3–4). **i** Cytochrome-c levels in the midbrain expressed as ng/mg total protein (*n* = 3–4). **j** 4-Hydroxy-2-nonenal levels in the midbrain expressed as ng/mg total protein (*n* = 3–4). **k** Cytochrome-c levels in the midbrain expressed as ng/mg total protein (*n* = 3). **l** 4-Hydroxy-2-nonenal levels in the midbrain expressed as ng/mg total protein (*n* = 3). **m** Oxidized LDL levels in the midbrain expressed as ng/mg total protein (*n* = 3–4). **n** Advanced glycation end product levels in the midbrain expressed as ng/mg total protein (*n* = 3–4). **o** Oxidized LDL levels in the midbrain expressed as ng/mg total protein (*n* = 3). **p** Advanced glycation end product levels in the midbrain expressed as ng/mg total protein (*n* = 3). **q** IL-6 levels in the midbrain expressed as pg/mg total protein (*n* = 3–4). **r** IL-17A levels in the midbrain expressed as pg/mg total protein (*n* = 3–4). **s** IL-6 levels in the midbrain expressed as pg/mg total protein (*n* = 3). **t** IL-17A levels in the midbrain expressed as pg/mg total protein (*n* = 3). **u** TNF-α levels in the midbrain expressed as pg/mg total protein (*n* = 3–4). **v** TNF-α levels in the midbrain expressed as pg/mg total protein (*n* = 3). Samples were obtained at days 32 and 62, respectively. Data are representative of one experiment. Protein levels were normalized to total protein concentration. RT-qPCR gene expression was normalized to *β-actin* and *Gapdh*. Significant difference in RT-qPCR experiments for CPT1A P479L experiment; Hash (#) significant difference between Wt+V and Wt+R, asterisk (*) significant difference between Wt+R and Cpt1a+R. Significant differences in RT-qPCR experiments in etomoxir study: ^#^significant difference between Wt+V and Wt+R+V, *significant difference between Wt+R+V and Wt+R+E. Significant differences for protein experiments; *p ≤ 0.05; **p ≤ 0.01; ***p ≤ 0.001; ****p ≤ 0.0001. Wt wildtype, P479L *Cpt1a* P479L homozygote genotype, E Etomoxir, R rotenone, V Vehicle. Center line = mean, and whiskers = standard error of the mean. Statistics: two-way or one-way ANOVA with a Tukey post hoc test.

Other hallmarks of PD are the downregulation of tyrosine hydroxylase (TH) in the striatum and the general deposition of α-syn in the brain^1,31^. We therefore assessed normalized TH protein concentrations in the midbrain using an enzyme-linked immunosorbent assay (ELISA) and found that P479L+R mice had significantly higher mean concentrations (0.35 ± 0.05 SEM, *p* = 0.03) compared to Wt+R mice (0.19 ± 0.03 SEM) (Fig. 3a). We also analyzed the normalized concentration of α-syn in the midbrain and found that Wt+R mice had significantly higher concentrations of α-syn (55.1 ± 6.74 SEM, *p* = 0.05) compared to Wt+V mice (26 ± 3.17 SEM), and this was not the case for the P479L+R mice (34.16 ± 9.16 SEM) (Fig. 3b). Based on these findings, we also evaluated the levels of dopamine in the midbrain. We found that rotenone resulted in the depletion of dopamine, and that Wt+R mice had significantly (*p* = 0.03) lower mean concentrations (0.05 ± 0.01 SEM) compared to P479L+R mice (0.27 ± 0.07 SEM) (Fig. 3e).

Following the differences in motor and non-motor behavior, and in serum levels of glucose, TH, α-syn, and dopamine levels in the midbrain between Wt+R, and P479L+R mice, we examined the effect of the CPT1A P479L mutation on the gene expression levels of select targets in the midbrain of these mice using reverse transcriptase quantitative polymerase chain reaction (RT-qPCR). The select targets were related to metabolic pathways (*Cpt1a*, *Cpt1c* and *Pgc1α*), oxidative stress (*Nox2*) and markers of reactive microglia/macrophage/astrocyte (*Iba1*, *Cd68* and *Gfap*) (Fig. 3f). Wt+R had a significantly (*p* = 0.05) higher normalized *Cpt1a* expression (1.51 ± 0.19 SEM) compared to Wt+V mice (1 ± 0.11 SEM). In addition, Wt+R mice had a significantly (*p* = 0.04) higher normalized expression of *Cd68* in the midbrain (1.22 ± 0.06 SEM) compared to Wt+V (1 ± 0.02 SEM). Furthermore, Wt+R mice had a significantly (*p* = 0.002) higher gene expression of *Iba1* (1.26 ± 0.05 SEM) compared to Wt+V (1 ± 0.04 SEM) and P479L+R mice (1.03 ± 0.04 SEM, *p* = 0.003). Finally, Wt+R mice had a significantly (*p* = 0.05) lower mean expression of *Pgc1α* (0.92 ± 0.02 SEM) compared to P479L+R mice (1.15 ± 0.05 SEM). In summary, this indicated that rotenone caused upregulation of *Cpt1a* expression, as also previously published^12,13^ and activated CNS resident microglia/macrophages. In addition, the activation of *Iba1*-expressing cells were attenuated by the P479L mutation. The higher normalized expression of *Pgc1α* in P479L+R mice could indicate that the mutation supported the mitochondrial biogenesis following rotenone exposure.

Based on the differences in normalized gene expression between the groups in the midbrain, we evaluated whether mitochondrial function (oxidative stress) and inflammation was affected at the protein level. The level of oxidative stress was evaluated based on the concentration of normalized oxidative stress biomarkers cytochrome-c (Cyt-c) and 4-hydroxy-2-nonenal (4-HNE), respectively. Cyt-c promotes oxidative stress and apoptosis whereas 4-HNE is a major product of lipid peroxidation^38^. Rotenone resulted in significant (*p* = 0.002) higher mean concentrations of Cyt-c in Wt (4.9 ± 0.24 SEM) but not in P479L mutant mice (± SEM) midbrains (Fig. 3i). This was accompanied by significantly higher levels of 4-HNE (*p* = 0.003) (Fig. 3j) and oxidized LDL (*p* = 0.04) (ox-LDL) (Fig. 3m) in the Wt+R (22.18 ± 2.82 SEM, 6.54 ± 0.81 SEM)) compared to P479L+R group (10.23 ± 0.94 SEM, 3.76 ± 0.33 SEM). This indicated increased oxidative stress in the rotenone-exposed Wt group. High levels of glucose can result in the production of advanced glycation end products (AGEs), which may result in protein oligomerization, oxidative stress and a pathological inflammatory response^39^. Therefore, we speculated whether the difference seen in glucose levels between the groups (Fig. 2a) could have affected the levels of AGEs in the midbrain. Interestingly, Wt mice exposed to rotenone (6.69 ± 1.2 SEM) had significantly (*p* = 0.03) higher levels of AGEs in the midbrain compared to P479L+R mice (3.26 ± 0.22 SEM) (Fig. 3n). Finally, we evaluated differences in the inflammatory cytokines IL-6, IL-17A and TNF-α, all of which are associated with in vivo models mimicking some aspects of PD. P479L+R mice had significantly lower concentrations of the aforementioned (5.67 ± 0.94 SEM, 63.49 ± 3.03 SEM, 22.59 ± 3.59 SEM) compared to Wt+R mice (17.58 ± 1.6 SEM *p* = 0.03, 107.3 ± 13.16 SEM *p* = 0.02, 46.39 ± 7.73 SEM *p* = 0.04) (Fig. 3q–r, u). In sum, these data indicate that the downregulation of CPT1A activity results in resistance to rotenone exposure due to lower levels of rotenone-induced oxidative stress and inflammation.

### Application of a CPT1 blocker in the chronic rotenone mouse models ameliorates diseased motor and non-motor behavior during continuous disease induction

To test if inhibition of CPT1, and not only the CPT1A isoform, could be used to ameliorate the chronic rotenone-induced disease phenotype in animals mimicking some aspects of PD-like disease, we inhibited CPT1 activity using the CPT1 antagonist etomoxir. After rotenone exposure for 32 days and verified pathological motor and non-motor behavior, the mice were randomized into two groups (Supplementary Fig. 1 for baseline before treatment start) receiving either etomoxir (Wt+R+E) or vehicle (Wt+R+V) alternating with rotenone (Fig. 1b). Mice received 18 administrations of etomoxir or vehicle treatment between day 32 and until day 60, as previously described^32^ (see method section for further information). The effect of pharmacological inhibition of CPT1 with etomoxir was evaluated through sensorimotor test (beam test at day 42) and motor test at day 60 (rotarod). Mice treated with etomoxir performed better in all tests compared to mice treated with vehicle (Fig. 1e, f). Wt+R+E mice took significantly less time to transverse the 0.6 cm beam (11.84 s ± 0.65 SEM, *p* = 0.03) compared to Wt+R+V at day 42 (15.22 s ± 1.08 SEM) (Fig. 1f). Wt+R+E mice had a significantly higher mean RPM (29 ± 1.98 SEM) (Fig. 1e) at day 60 compared to Wt+R+V mice (22.33 ± 1.23 SEM, *p* = 0.013). In addition, we previously found that etomoxir resulted in increased activity in the cylinder test and increased normalized grip strength compared to day 32^32^. Taken together this indicated that inhibition of CPT1 attenuated impaired motor and sensorimotor functions.

### Etomoxir decreases the LDL serum concentration and increases the LDL/HDL ratio in rotenone-exposed mice

Rotenone treatment resulted in increased serum levels of glucose, and P479L+R mice showed a decrease in serum glucose levels (Fig. 2a). Accordingly, we hypothesized that etomoxir could affect serum glucose levels in the chronic rotenone-exposed mice. Our results showed that Wt+R+V mice had a significantly higher serum glucose level at day 62 (11.79 ± 1.44 SEM) compared to Wt+V mice (3.31 ± 044 SEM, *p* = 0.005) (Fig. 2c), and this could not be attributed to differences in weight (Fig. 1i). Etomoxir-treated mice did not have a significantly higher glucose level compared to Wt+V mice, and we did not find a statistically significant difference between vehicle and etomoxir-treated mice. LDL-c and oxidation of LDL-c increases the activity of CPT1, stimulates inflammation and production of mitochondrial reactive oxygen species^37^ and is associated with PD^40^. Thus, we evaluated whether the downregulation of CPT1 activity by etomoxir affected serum levels of LDL-c. We found that etomoxir resulted in a shift toward a healthy lipoprotein profile compared to Wt+R+V mice characterized by lower LDL-c (Wt+R+E: 0.99 ± 0.06 SEM, Wt+R+V: 2.26 ± 0.37 SEM, *p* = 0.04) (Fig. 2d), higher HDL-c (Wt+R+E 1.02 ± 0.11 SEM, Wt+R+V 0.64 ± 0.056 SEM, *p* = 0.04) (Fig. 2g) and decreased LDL-c/HDL-c ratio (Wt+R+E: 1.02 ± 0.18 SEM, Wt+R+V: 3.47 ± 0.31 SEM, *p* = 0.001) (Fig. 2h). This indicates that the inhibition of CPT1 modulates lipoprotein metabolism, as previously described^37^.

### Etomoxir attenuates protein concentrations and normalized gene expression of PD biomarkers in the midbrain

The behavior tests indicated disease amelioration and we therefore assessed the level of TH in the striatum of the mice using western blot. We found that W+R+E (0.94 ± 0.07 SEM, *p* = 0.05) and Wt+V mice (1 ± 0.7 SEM, *p* = 0.02) had significantly higher TH protein concentrations compared to Wt+R+V mice (0.66 ± 0.08 SEM) (Supplementary Fig. 2). This was further confirmed using sandwich ELISA (Fig. 3c). Next, we investigated differences in the normalized concentrations of α-syn in the midbrain using sandwich ELISA. We found that rotenone resulted in increased levels of α-syn (53.72 ± 8.43 SEM) and this was fully rescued using etomoxir (22.68 ± 1.74 SEM, *p* = 0.01) (Fig. 3d). Rotenone results in the death of DA neurons, and thereby depletion of dopamine^1^. However, we did not find any significant differences between the normalized concentrations of dopamine in the midbrain following etomoxir treatment when comparing Wt+V or Wt+R+E to Wt+R+V mice (Fig. 3g).

We also evaluated changes in normalized gene expression of metabolic, oxidative stress and reactive microglia/macrophage markers in the midbrain (Fig. 3h). These results showed that Wt+R+V mice had a significantly higher expression of *Nox2* (1.78 ± 0.25 SEM, *p* = 0.02) and *Iba1* (1.23 ± 0.05 SEM, *p* = 0.05) compared to Wt+V (*Nox2* 1.0 ± 0.09 SEM, *Iba1* 1.03 ± 0.02 SEM), while this was not the case for Wt+R+E (*Nox2* 1.35 ± 0.09 SEM, *Iba1* 1.07 ± 0.08 SEM). Furthermore, Wt+R+V mice had significantly lower gene expression of *Pgc1α* (0.78 ± 0.13 Range) compared to Wt+R+E mice (1.04 ± 0.9 range, *p* = 0.04), indicating that rotenone increased expression of genes that are considered markers of oxidative stress and activation of reactive microglia/macrophages. The downregulation of CPT1 activity counteracted the expression of markers to some extent.

Based on the differences in normalized gene expression between the groups in the midbrain, we evaluated whether mitochondrial function (oxidative stress) and inflammation was affected at the protein level. First, we evaluated whether the downregulation of CPT1 using etomoxir affected mitochondrial dysfunction based on the normalized concentration of Cyt-c in the midbrain. We found that etomoxir resulted in a significantly lower concentration of Cyt-c (2.69 ± 0.22 SEM) compared to Wt+R+V mice (4.3 ± 0.38 SEM, *p* = 0.01) (Fig. 3k) which supported that etomoxir diminished mitochondrial dysfunction, and that this was accompanied by significant lower normalized concentrations of markers of oxidative stress (Fig. 3l, o). Furthermore, etomoxir resulted in significantly lower normalized concentrations of AGEs and pro-inflammatory cytokines (IL-6, IL-17A and TNF-α) in the midbrain (Fig. 3p, s, t, v). In general, these findings indicated that the downregulation of CPT1 activity by etomoxir was effective in ameliorating rotenone-induced disease characteristics mimicking some aspects of PD.

### Application of a CPT1 blocker in the chronic rotenone mouse models ameliorates non-motor impairment during a washout period

**Fig. 4:**
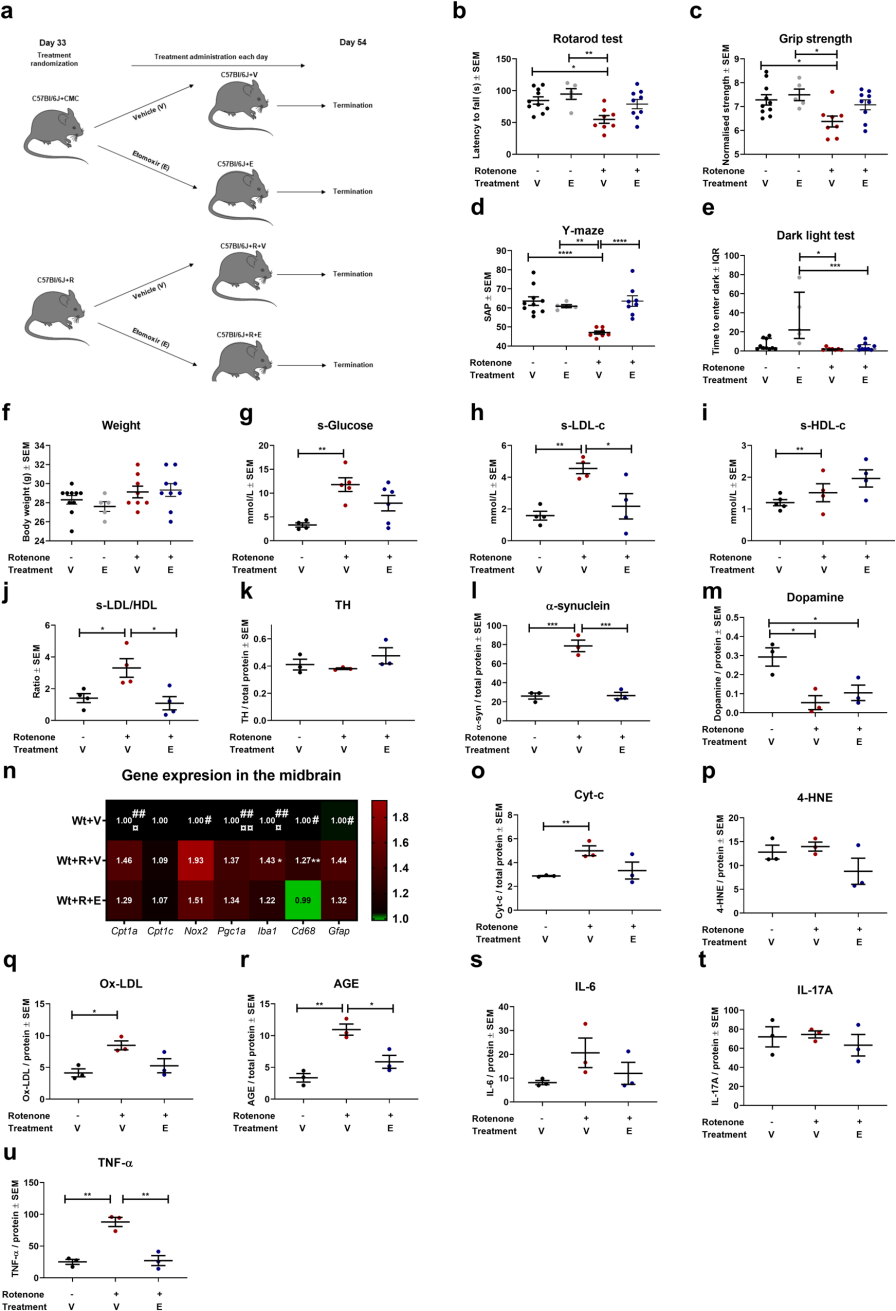
Downregulation of CPT1 activity by etomoxir ameliorates rotenone-induced cognitive impairment during and following a washout period and modulates gene expressions in the midbrain. **a** Male C57Bl/6J and mice received vehicle (carboxymethylcellulose sodium salt, 0.5%) or rotenone (30 mg/kg) for 32 days followed by daily treatment with etomoxir or vehicle until termination at day 54. **b** Mean latency to fall off the rotarod test at day 54 (*n* = 5–10). **c** Mean normalized grip strength at day 54 (*n* = 5–10). **d** Visuospatial memory expressed as mean spontaneous alternation percentage in the Y-maze test at day 54 (*n* = 5–10). **e** Median time to enter dark in the dark-light box test at day 54 (*n* = 5–10). **f** Mean weight of mice at day 32 in grams (*n* = 5–10). **g** Mean serum glucose levels measured in mmol/l (*n* = 4–6). **h** Mean serum LDL-c levels measured in mmol/l (*n* = 4). **i** Mean serum HDL-c levels measured in mmol/l (*n* = 4). **j** Mean ratio of serum LDL-c/HDL-c levels (*n* = 4). **k** TH levels in the midbrain expressed as mean ng/mg total protein (*n* = 3). **l** α-Synuclein levels in the midbrain expressed as mean pg/mg total protein (*n* = 3). **m** Dopamine levels in the midbrain expressed as mean ng/mg total protein (*n* = 3). **n** Heatmap illustrating mean fold gene expression change in the midbrain of *Cpt1a, Cpt1c, Nox2, Pgc1a, Iba1, Cd68* and *Gfap* (*n* = 4–5). **o** Cytochrome-c levels in the midbrain expressed as ng/mg total protein (*n* = 3). **p** 4-Hydroxy-2-nonenal levels in the midbrain expressed as ng/mg total protein (*n* = 3). **q** Oxidized LDL levels in the midbrain expressed as ng/mg total protein (*n* = 3). **r** Advanced glycation end product levels in the midbrain expressed as ng/mg total protein (*n* = 3). **s** IL-6 levels in the midbrain expressed as pg/mg total protein (*n* = 3). **t** IL-17A levels in the midbrain expressed as pg/mg total protein (*n* = 3). **u** TNF-α levels in the midbrain expressed as pg/mg total protein (*n* = 3). Serum samples and brains were obtained at day 54. Error bars represent the standard error of the mean (SEM) or interquartile rate of the median (IQR). Data are representative of one experiment. Protein levels (TH, α-synuclein, and dopamine) were normalized to total protein concentration. RT-qPCR gene expression was normalized to *β-actin* and *Gapdh*. Significant differences for behavior, serum and protein experiments; *p ≤ 0.05; **p ≤ 0.01; ***p ≤ 0.001; ****p ≤ 0.0001. Significant differences in RT-qPCR experiments; #=significant difference between Wt+V and Wt+R+V, ¤significant difference between Wt+V and Wt+R+E, *significant difference between Wt+R+V and Wt+R+E. Wt wildtype, R rotenone, V Vehicle, E etomoxir, SAP spontaneous alternation percentage, TH tyrosine hydroxylase. Center line = mean, and whiskers = standard error of the mean except for **e**, center line = median and whiskers = interquartile range. Statistics: one-way ANOVA followed by Tukey post hoc test and Kruskal–Wallis test followed by Dunn post hoc test except for panels **b**–**f**, which were analyzed by two-way ANOVA followed by Tukey post hoc. We acknowledge Servier Medical Art for the mouse illustration, with the following license: https://creativecommons.org/licenses/by/3.0/. No changes were made to the drawing.

It has been shown that the level of TH-positive DA neurons in vitro is acutely decreased upon rotenone exposure but restored to some extent following a washout period^41^. Hence, we speculated whether some of the effects observed in the alternating rotenone etomoxir experiment were due to the continuous administration of rotenone. We hypothesized that etomoxir could also be effective during and following a washout period. Therefore, C57Bl/6J male mice were randomized into daily treatment with etomoxir (Wt+R+E) or vehicle (Wt+R+V) following 32 days of rotenone exposure and no further administration (Supplementary Fig. 3 for baseline before treatment start). In addition, non-rotenone-exposed C57Bl/6J mice were divided into treatment with vehicle (Wt+V) or etomoxir (Wt+E) (Fig. 4a). Mice received daily treatment for 21 days, and at day 54, the mice were tested for impaired motor behavior using rotarod and grip strength tests. We observed significant differences between Wt+R+V (mean latency to fall 54.84 ± 6.13 SEM, mean grip strength 6.37 ± 0.23 SEM) and healthy Wt+V controls (mean latency to fall 84.57 ± 5.78 SEM, *p* = 0.01 mean grip strength 7.28 ± 0.22 SEM, *p* = 0.03) (Fig. 4b, c) but no differences between Wt+R+V and Wt+R+E. This indicated that the inhibition of CPT1 potentially had some effect on the motor impairment but not enough to restore motor function compared to healthy controls. We also evaluated non-motor behavior as PD is characterized by non-motor symptoms such as cognitive impairment and anxiety^1^. Wt+R+E mice had a significantly higher spontaneous alternation percentage in the Y-maze test (63.58 ± 2.76 SEM) compared to Wt+R+V mice (47.05 ± 0.78 SEM, *p* = 0.0001) (Fig. 4d). Hence, the downregulation of CPT1 activity resulted in amelioration of cognitive impairment (Fig. 1j). Finally, we evaluated whether administration of etomoxir affected anxiety-like behavior following the washout period. However, both Wt+R+V and Wt+R+E mice had a significantly lower time to enter the dark in the dark-light box test compared to Wt+E mice, indicating no effect on anxiety-like behavior (Fig. 4e).

### Application of a CPT1 blocker in the chronic rotenone mouse model results in a decreased LDL concentration and increased LDL/HDL ratio during a washout period

As in the previous experiments, we also evaluated the serum levels of glucose. Etomoxir treatment following rotenone exposure did not result in a significantly lower serum glucose level compared to Wt+R+V (Fig. 4g). However, etomoxir did result in a significantly lower concentration of LDL (2.17 ± 0.79 SEM) and lower LDL-c/HDL-c ratio (1.09 ± 0.41 SEM) in the serum compared to Wt+R+V (4.56 ± 0.33 SEM, *p* = 0.03, 3.31 ± 0.59 SEM, *p* = 0.02) (Fig. 4h–j). These results indicated that inhibition of CPT1 restored the lipoprotein profiles toward normal homeostasis in accordance with the finding in Fig. 2, which might affect inflammation, oxidative stress and possibly α-syn deposition, as previously described^36,37,40^.

### Application of a CPT1 blocker in the chronic rotenone mouse model attenuates pathological protein concentrations and normalized gene expression levels of inflammatory activity during a washout period

As in the experiments described above, we evaluated the normalized concentrations of TH, α-syn, and dopamine in the midbrain. We did not find any differences in TH levels between the groups (Fig. 4k). However, the healthy mice receiving vehicle (26.02 ± 3.17 SEM, *p* = 0.0004) and Wt+R+E mice (26.53 ± 3.4 SEM, *p* = 0.0004) had significantly lower levels of α-syn compared to Wt+R+V mice (78.65 ± 6.05 SEM) (Fig. 4l). In addition, the mice exposed to rotenone had significantly lower dopamine levels compared to non-exposed mice (Fig. 4m).

Next, we evaluated whether downregulation of CPT1 activity by etomoxir during the washout period affected normalized gene expression of metabolic, oxidative stress and reactive microglia/macrophage markers in the midbrain. Rotenone resulted in significant upregulation of *Cpt1a* gene expression in the midbrain (Fig. 4n), as previously described in vitro^12^. In addition, we found that Wt+R+V mice had significantly higher normalized gene expression of *Nox2* (1.93 ± 0.25 SEM, *p* = 0.02), *Iba1* (1.43 ± 0.04 SEM, *p* = 0.0001), *Cd68* (1.27 ± 0.8 SEM, *p* = 0.01) and *Gfap* (1.43 ± 0.83 range, *p* = 0.02) compared to Wt+V mice (*Nox2* 1.0 ± 0.09 SEM, *Iba1* 1 ± 0.03 SEM, *Cd68* 1 ± 0.03 SEM, *Gfap* 1 ± 0.28 range) (Fig. 4n). This illustrates that rotenone exposure, promotes sustained pathological marker genes upregulation even following a washout period. Wt+R+E mice had significantly lower expression of *Iba1* (1.22 ± 0.05 SEM, *p* = 0.01) and *Cd68* (0.98 ± 0.02 SEM, *p* = 0.009), compared to Wt+R+V mice, which indicated that downregulation of CPT1 activity resulted in modulation of the activation of reactive microglia/macrophages in the midbrain (Fig. 4n).

Based on the changes in gene expression, we investigated whether mitochondrial dysfunction, oxidative stress and inflammation were affected at the protein level. Rotenone resulted in significantly higher normalized concentrations of Cyt-c indicating that mitochondrial dysfunction was still present following the washout period (Fig. 4o), and this was associated with higher concentrations of Ox-LDL in the Wt+R+V mice (8.47 ± 0.69 SEM,) compared to Wt+V (4.14 ± 0.63 SEM, *p* = 0.02) (Fig. 4q). However, we did not find any difference in 4-HNE levels between the groups (Fig. 4p). The Wt+R+V mice had significantly higher levels of AGEs (10.95 ± 0.88 SEM) in the midbrain compared to both healthy controls (3.37 ± 0.67 SEM, *p* = 0.002) and Wt+R+E (5.88 ± 1.02 SEM, *p* = 0.02) (Fig. 4r) which may be associated with the higher glucose levels. Finally, we investigated whether etomoxir diminished pro-inflammatory cytokine concentrations (IL-6, IL-17A and TNF-α). There was no difference in the IL-6 and IL-17A levels (Fig. 4s, t) but etomoxir resulted in significantly lower levels of TNF-α (27.1 ± 7.81 SEM) in the midbrain compared to Wt+R+V mice (87.94 ± 7.21 SEM, *p* = 0.002) (Fig. 4u). In general, the results indicated that inhibition of CPT1 during and following a washout period resulted in diminished memory impairment and, to some degree, attenuated inflammation.

### PARK2 mutant mice present with pathological behavior disrupted glucose metabolism and pathological gene expression in the midbrain

**Fig. 5:**
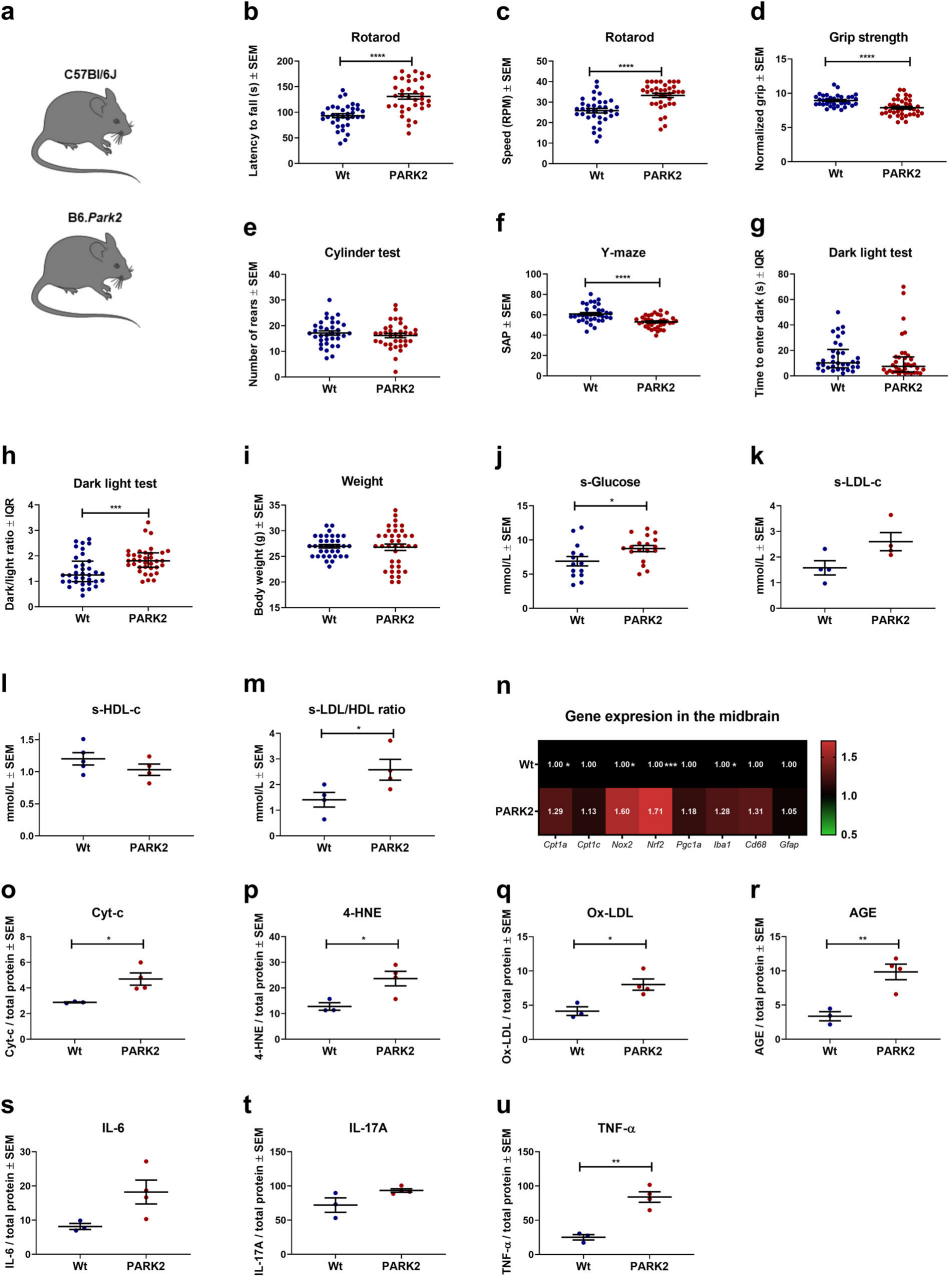
PARK2 knockout is associated with impaired motor and non-motor functions, disrupted glucose and lipoprotein metabolism and pathological gene expressions in the midbrain. **a** Male C57Bl/6J mice and male PARK2 knockout male mice on C57Bl/6J background were compared. **b** Mean latency to fall of the rotarod (*n* = 35–36). **c** Mean rounds per minute on the rotarod (*n* = 35–36). **d** Mean normalized grip strength (*n* = 35–37). **e** Spontaneous activity in the cylinder test expressed as a mean number of rears (*n* = 35–36). **f** Visuospatial memory expressed as mean spontaneous alternation percentage in the Y-maze test (*n* = 35–37). **g** Median time to enter dark in the dark-light box test (*n* = 34–37). **h** Median dark/light ratio in the dark-light box test (*n* = 35–36). **i** Mean weight of mice at day 32 in grams (*n* = 35–37). **j** Mean serum glucose levels measured in mmol/l (*n* = 14–18). **k** Mean serum LDL-c levels measured in mmol/l (*n* = 4). **l** Mean serum HDL-c levels measured in mmol/l (*n* = 4–5). **m** Mean ratio of serum LDL-c/HDL-c levels (*n* = 4). **n** Heatmap illustrating mean fold gene expression change in the midbrain of *Cpt1a, Cpt1c, Nox2, Nrf2, Pgc1a, Iba1, Cd68* and *Gfap* (*n* = 4–5). **o** Cytochrome-c levels in the midbrain expressed as ng/mg total protein (*n* = 3–4). **p** 4-Hydroxy-2-nonenal levels in the midbrain expressed as ng/mg total protein (*n* = 3–4). **q** Oxidized LDL levels in the midbrain expressed as ng/mg total protein (*n* = 3–4). **r** Advanced glycation end product levels in the midbrain expressed as ng/mg total protein (*n* = 3–4). **s** IL-6 levels in the midbrain expressed as pg/mg total protein (*n* = 3–4). **t** IL-17A levels in the midbrain expressed as pg/mg total protein (*n* = 3–4). **u** TNF-α levels in the midbrain expressed as pg/mg total protein (*n* = 3–4). Serum samples and brains were obtained at the same time points. Error bars represent the standard error of the mean (SEM) or interquartile rate of the median (IQR). Data are representative of one experiment. RT-qPCR gene expression was normalized to *β-actin* and *Gapdh*. Significant differences for behavior, serum and RT-qPCR experiments; *p ≤ 0.05; **p ≤ 0.01; ***p ≤ 0.001; ****p ≤ 0.0001. PARK2 PARK2 knockout mice, Wt wildtype, RPM rounds per minute, SAP spontaneous alternation percentage. Center line = mean, and whiskers = standard error of the mean except for **g** and **h**, center line = median and whiskers = interquartile range. Statistics: unpaired two-tailed *t*-test or Mann–Whitney *U* test. We acknowledge Servier Medical Art for the mouse illustration, with the following license; https://creativecommons.org/licenses/by/3.0/. No changes were made to the drawing.

PD patients with PARK2 mutations have upregulated β-oxidation and dysregulated glucose metabolism^14,15^. In conjunction with this, we hypothesized that the inhibition of CPT1 could be effective in a PARK2 knockout mouse model mimicking the autosomal recessive form of familiar PD^42^. Therefore, we first evaluated whether male PARK2 mice had any motor and non-motor abnormalities compared to male C57Bl/6J mice (Wt) (Fig. 5a). PARK2 mutated mice performed significantly better in the rotarod tests compared to Wt mice (Fig. 5b, c), which could be due to the increased extracellular dopamine levels in this in vivo model^42^. However, PARK2 mice had significantly lower muscle strength (5.77 ± 0.2 SEM) compared to Wt mice (7.6 ± 0.14 SEM, *p* = 0.0001) (Fig. 5d). This indicated potential pathological muscle function. We did not observe any differences in spontaneous activity in the cylinder test (Fig. 5e). Following the evaluation of motor behavior, we investigated whether PARK2 mice had significant differences in non-motor behavior compared to Wt mice. We found that PARK2 mice had significantly lower spontaneous alternation percentage in the Y-maze test (53.1 ± 0.95 SEM) compared to Wt mice (60.82 ± 1.37 SEM, *p* = 0.0001) (Fig. 5f), which pointed toward memory impairment due to the PARK2 mutation. In addition, we speculated whether the disrupted metabolism due to the mutation could affect anxiety-like behavior. PARK2 mice and Wt mice had no difference in time to enter the dark in the dark-light box test (Fig. 5g), but PARK2 mice spent a significantly longer time in the dark (1.8 ± 2.34 range) compared to Wt mice (1.25 ± 2.22 range, *p* = 0.0008) (Fig. 5h). This indicated that PARK2 mice had anxiety-like behavior. Therefore, overall PARK2 mice presented with some impaired motor (decreased grip strength) and non-motor (Y-maze and dark-light test) functions mimicking symptoms seen in PD patients^1^.

Next, we investigated whether PARK2 mice had any differences in glucose and lipoprotein levels in serum compared to Wt mice. We found that PARK2 mice had significantly higher concentrations of glucose (8.75 ± 0.45 SEM) (Fig. 5j) and an increased LDL-c/HDL-c ratio (2.58 ± 0.41 SEM) compared to Wt mice (mean glucose 6.9 ± 0.69 SEM, *p* = 0.03, mean LDL-c/HDL-c ratio 1.41 ± 0.28 SEM, *p* = 0.05) (Fig. 5k–m), which could not be explained by differences in weight (Fig. 5i). This illustrated that the PARK2 knockout model mimicked some biochemical alternations seen in PD^5,36,40^ and associated with pathological processes such as inflammation and oxidative stress^36,37^.

We also investigated whether PARK2 mice had any changes in normalized gene expression of β-oxidation, oxidative stress, and reactive microglia/macrophage markers in the midbrain. PARK2 mice had significantly increased expression of *Cpt1a* (1.29 ± 0.07 SEM, *p* = 0.02), *Nox2* (1.6 ± 0.22 SEM, *p* = 0.05), *Nrf2* (1.71 ± 0.08 SEM, *p* = 0.0002) and *Iba1* (1.28 ± 0.06 SEM, *p* = 0.006) (Fig. 5n) in the midbrain compared to Wt mice (*Cpt1a* 1 ± 0.6 SEM, *Nox2* 1 ± 0.9 SEM, *Nrf2* 1 ± 0.03 SEM, *Iba1* 1 ± 0.03 SEM).

Finally, we investigated whether there was any difference in protein levels of select targets related to mitochondrial dysfunction, oxidative stress and inflammation. PARK2 mice had significantly higher normalized concentrations of Cyt-c (4.69 ± 0.48 SEM, *p* = 0.03), 4-HNE (23.64 ± 2.84 SEM, *p* = 0.03), Ox-LDL (8.02 ± 0.82 SEM, *p* = 0.02), AGEs (9.84 ± 1.13 SEM, *p* = 0.007) and TNF-α (83.89 ± 7.7 SEM, *p* = 0.002) compared to Wt mice (Cyt-c 2.88 ± 0.38 SEM, 4-HNE 12.79 ± 1.46 SEM, Ox-LDL 4.14 ± 0.63 SEM, AGEs 3.37 ± 0.67 SEM, TNF-α 25.14 ± 4.0 SEM) (Fig. 5o–r, u). However, we did not find any difference in IL-6 and IL-17A levels (Fig. 5s, t). This indicates that the PARK2 knockout model is characterized by increased β-oxidation, mitochondrial dysfunction, oxidative stress, reactive microglia/macrophages, and inflammation in the midbrain. However, the PARK2 knockout mouse model is not characterized by decreased levels of dopamine nor accumulation of α-syn in the midbrain^42^. This was confirmed by our data (Supplementary Fig. 4), Accordingly, PARK2 PD patients do not have an accumulation of α-syn aggregates in the CNS, but are characterized by mitochondrial dysfunction, oxidative stress and inflammation^14,15,43,44^.

### Inhibition of CPT1 activity in PARK2 mutant mice improves muscle strength and non-motor behavior and diminishes pathological gene expression in the midbrain

**Fig. 6:**
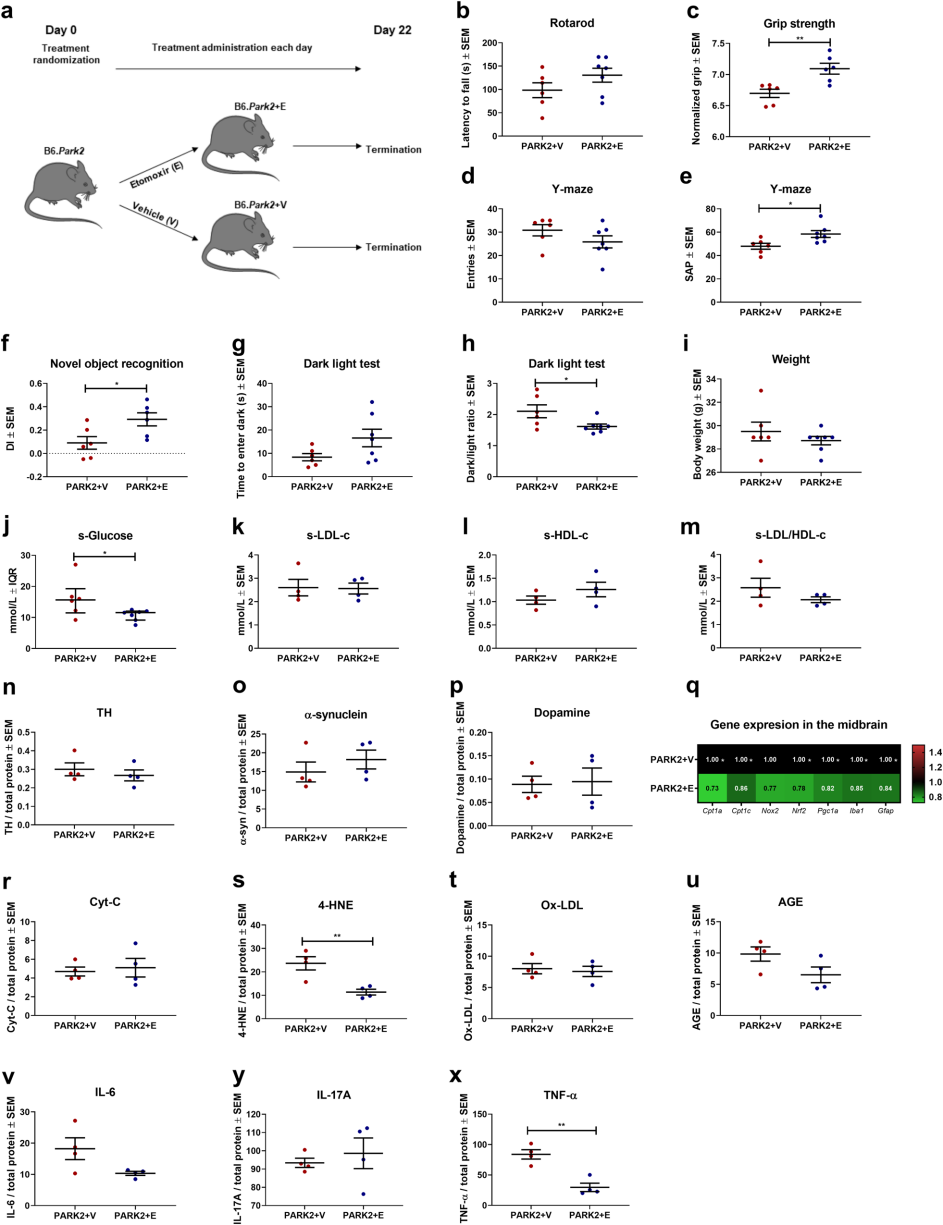
Inhibition of CPT1 activity by etomoxir attenuated impaired muscle function, non-motor behavior and diminished pathological gene expression in the midbrain in PARK2 knockout mice. **a** Male PARK2 knockout mice on C57Bl/6J background were randomized into treatment with etomoxir or vehicle for 22 days. **b** Mean latency to fall of the rotarod (*n* = 6–7). **c** Mean normalized grip strength (*n* = 6). **d** Spontaneous activity in the Y-maze expressed as mean number of entries (*n* = 6–7). **e** Visuospatial memory expressed as mean spontaneous alternation percentage in the Y-maze test (*n* = 6–7). **f** Object recognition memory expressed as mean discrimination index in the novel object recognition test (*n* = 6). **g** Mean time to enter dark in the dark-light box test (*n* = 6–7). **h** Mean dark/light ratio in the dark-light box test (*n* = 6–7). **i** Mean weight of mice at day 32 in grams (*n* = 6–7). **j** Median serum glucose levels measured in mmol/l (*n* = 6–7). **k** Mean serum LDL-c levels measured in mmol/l (*n* = 4). **l** Mean serum HDL-c levels measured in mmol/l (*n* = 4). **m** Mean ratio of serum LDL-c/HDL-c levels (*n* = 4). **n** TH levels in the midbrain expressed as mean ng/mg total protein (*n* = 4). **o** α-Synuclein levels in the midbrain expressed as mean pg/mg total protein (*n* = 4). **p** Dopamine levels in the midbrain expressed as mean ng/mg total protein (*n* = 4). **q** Heatmap illustrating mean normalized fold gene expression change in the midbrain of *Cpt1a, Cpt1c, Nox2, Nrf2, Pgc1a, Iba1, Gfap* and *Cd68* (*n* = 4–5). **r** Cytochrome-c levels in the midbrain expressed as ng/mg total protein (*n* = 4). **s** 4-Hydroxy-2-nonenal levels in the midbrain expressed as ng/mg total protein (*n* = 4). **t** Oxidized LDL levels in the midbrain expressed as ng/mg total protein (*n* = 4). **u** Advanced glycation end product levels in the midbrain expressed as ng/mg total protein (*n* = 4). **v** IL-6 levels in the midbrain expressed as pg/mg total protein (*n* = 4). **y** IL-17A levels in the midbrain expressed as pg/mg total protein (*n* = 4). **x** TNF-α levels in the midbrain expressed as pg/mg total protein (*n* = 4). Animals were tested at day 22 and serum and brain samples were obtained at day 22. Error bars represent the standard error of the mean (SEM) or interquartile rate of the median (IQR). Data are representative of one experiment. Protein levels (TH, α-synuclein, and dopamine) were normalized to total protein concentration. RT-qPCR gene expression was normalized to *β-actin* and *Gapdh*. Significant differences for behavior, serum, protein and RT-qPCR experiments; *p ≤ 0.05; **p ≤ 0.01; ***p ≤ 0.001; ****p ≤ 0.0001. PARK2 *Park2* knockout genotype, V Vehicle, E etomoxir, SAP spontaneous alternation percentage, DI discrimination index, TH tyrosine hydroxylase. Center line = mean, and whiskers = standard error of the mean except for **j**, center line = median and whiskers = interquartile range. Statistics: two-tailed unpaired *t*-test, Mann–Whitney *U* test. We acknowledge Servier Medical Art for the mouse illustration, with the following license: https://creativecommons.org/licenses/by/3.0/. No changes were made to the drawing.

Based on the findings that PARK2 knockout mice have motor, non-motor and gene expression changes in the midbrain, indicating increased CPT1A lipid metabolism, oxidative stress and inflammation, PARK2 mutated male mice were randomized into treatment with etomoxir (PARK2+E) or vehicle (PARK2+V) for 21 days (Fig. 6a) based on the C57Bl/6J etomoxir studies described above. First, we evaluated motor function and muscle strength. PARK2+E mice did not have a significantly higher latency to fall of the rotarod compared to PARK2+V mice (Fig. 6b) but etomoxir-treated mice had a significantly higher mean normalized grip strength (7.09 ± 0.09 SEM) compared to vehicle-treated mice (6.7 ± 0.07 SEM, *p* = 0.005) (Fig. 6c). This indicated that the downregulation of CPT1 activity did not affect the increased motor function but restored muscle strength. Next, we evaluated whether etomoxir treatment improved the non-motor symptoms observed in PARK2 mice. PARK2+E mice had significantly higher visuospatial memory (58.45 ± 2.94 SEM, *p* = 0.02) (Fig. 6e), discrimination index (DI) (0.29 ± 0.06 SEM, *p* = 0.03) (Fig. 6f), and decreased anxiety-like behavior (1.61 ± 0.08 SEM, *p* = 0.04) (Fig. 6g, h) compared to PARK2 mice receiving vehicle (49.97 ± 2.52 SEM, 0.09 ± 0.05 SEM, 2.12 ± 0.21 SEM respectively). This indicated that the downregulation of CPT1 activity attenuated the impaired non-motor functions observed in PARK2 mice.

Induced pluripotent stem cells from patients with *PARK2* knockout mutations have impaired glucose metabolism^15^. Based on this and the fact that PARK2 knockout mice have hyperglycemia compared to Wt mice, we evaluated the glucose levels in serum from the PARK2+V and PARK2+E mice. We found that PARK2 knockout mice treated with etomoxir had significantly lower glucose concentrations (11.55 ± 4.94 range) compared to PARK2+V mice (15.67 ± 17.78 range, *p* = 0.04) (Fig. 6j). This indicated that the downregulation of CPT1 restored glucose metabolism. Following this, we evaluated the concentrations of LDL-c, HDL-c, and LDL-c/HDL-c in serum. We did not find any significant differences in LDL-c, HDL-c or LDL/HDL ratio (Fig. 6k–m).

### Inhibition of CPT1 activity in PARK2 mutant attenuates pathological gene expression and TNF-α concentration in the midbrain

Multiple studies have reported that the PARK2 knockout mouse models are not associated with changes in the level of DA neurons in the midbrain^42,45^. This was consistent with findings from this study showing no changes in TH (Fig. 6n, p). *PARK2* patients rarely present with deposition of α-syn in the brain^43,46^. In accordance with this, we did not find any differences in the level of α-syn between the two treatment groups (Fig. 6o).

We found that PARK2 knockout mice have changes in normalized gene expression of metabolic, oxidative stress and inflammatory genes in the midbrain compared to Wt mice (Fig. 5n). Based on this, we evaluated whether etomoxir modulated the expression of these genes in the midbrain. PARK2+E mice had significantly lower expression of *Cpt1a* (0.73 ± 0.03 SEM), *Cpt1c* (0.86 ± 0.02 SEM), *Nrf2* (0.78 ± 0.07 SEM), *Pgc1α* (0.82 ± 0.3 SEM), *Iba1* (0.85 ± 0.3 SEM) and *Gfap* (0.84 ± 0.03 SEM) compared to PARK2+V (*Cpt1a* 1 ± 0.05 SEM, *p* = 0.004, *Cpt1c* 1 ± 0.04 SEM, *p* = 0.04, *Nrf2* 1 ± 0.5 SEM, *p* = 0.03, *Pgc1α* 1 ± 0.4 SEM, *p* = 0.01, *Iba1* 1 ± 0.05 SEM, *p* = 0.03, *Gfap* 1 ± 0.5 SEM, *p* = 0.04) (Fig. 6q). This indicates that downregulation of CPT1 activity resulted in lower normalized gene expression of markers of oxidative stress, reactive microglia/macrophages and reactive astrocytes. In accordance, *PARK2* knockout mutations result in increased oxidative stress based on the NOX2-NRF2 pathway in neurons^43^. In addition, induced macrophages from PARK2 knockout mice have increased levels of inflammatory cytokines and PARK2 knockout mice have increased susceptibility to inflammation-related DA neuron degeneration^47,48^.

Finally, we evaluated whether pharmacological downregulation of CPT1 affected the normalized protein concentrations of Cyt-c, 4-HNE, Ox-LDL, AGEs and the pro-inflammatory cytokines IL-6, IL-17A and TNF-α. Etomoxir did not affect the level of Cyt-c (Fig. 6r) but significantly decreased the level of 4-HNE (11.36 ± 1.23 SEM) compared to PARK2+V mice (23.64 ± 2.84 SEM, *p* = 0.007) (Fig. 6s). The downregulation did not modulate the level of Ox-LDL, AGEs, IL-6 or IL-17A (Fig. 6t–y) in the midbrain. However, etomoxir did result in a significantly lower level of TNF-α (29.68 ± 6.95 SEM) compared to vehicle-treated mice (83.89 ± 7.7 SEM, *p* = 0.002) (Fig. 6x). Overall, the results indicate that PARK2 knockout mice display impaired muscle strength and non-motor behavior and that downregulation of CPT1 activity ameliorates these symptoms, decreases hyperglycemia and diminishes oxidative stress, reactive microglia markers and inflammation in the midbrain based on normalized gene expression and protein concentrations.

### Rotenone exacerbates impaired motor and non-motor functions in PARK2 knockout mice

**Fig. 7:**
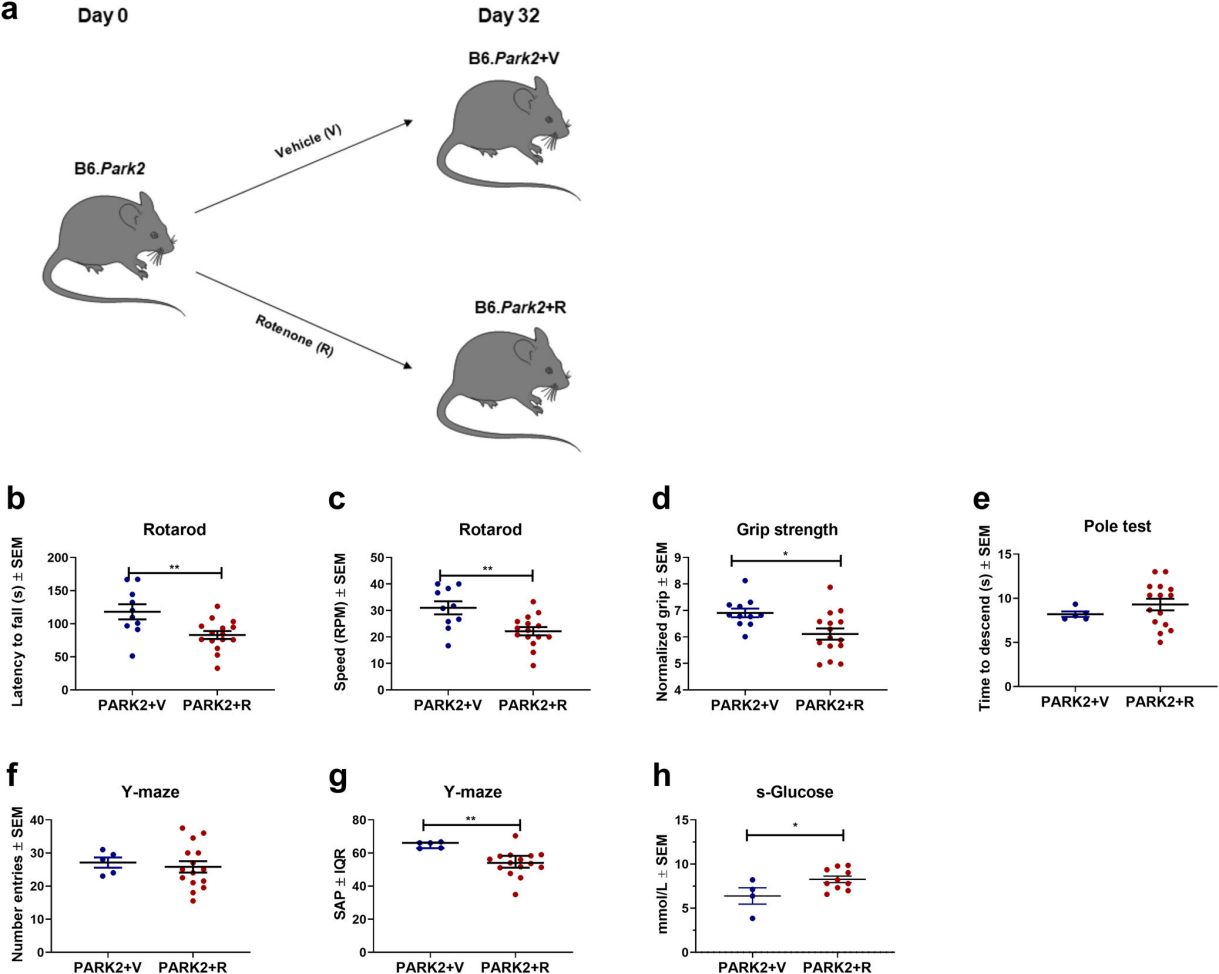
Rotenone aggravates motor and non-motor dysfunction in PARK2 knockout mice. **a** Male PARK2 knockout mice were randomized into rotenone (30 mg/kg) exposure or vehicle (carboxymethylcellulose sodium salt, 0.5%) for 32 days. **b** Mean latency to fall of the rotarod (*n* = 10–15). **c** Mean rounds per minute on the rotarod (*n* = 10–15). **d** Mean normalized grip strength (*n* = 11–15). **e** Mean time to descend the pole in the pole test (*n* = 5–15). **f** Spontaneous activity in the Y-maze expressed as mean number of entries (*n* = 5–15). **g** Visuospatial memory expressed as mean spontaneous alternation percentage in the Y-maze test (*n* = 5–15). **h** Mean serum glucose levels measured in mmol/l (*n* = 4–10). Animals were tested at day 32 and serum samples were obtained at day 32. Error bars represent the standard error of the mean (SEM). Data are representative of one experiment. Significant differences for behavior and serum experiments; *p ≤ 0.05; **p ≤ 0.01; ***p ≤ 0.001; ****p ≤ 0.0001. PARK2 PARK2 knockout genotype, R rotenone, V Vehicle, SAP spontaneous alternation percentage. Center line = mean, and whiskers = standard error of the mean except for **g**, center line = median and whiskers = interquartile range. Statistics: two-tailed unpaired *t*-test. We acknowledge Servier Medical Art for the mouse illustration, with the following license: https://creativecommons.org/licenses/by/3.0/. No changes were made to the drawing.

PARK2 knockout models have increased susceptibility to mitochondrial complex I blockade, and PARKIN overexpression protects against damage caused by complex I blockade^45,49,50^. Based on this and the fact that the PARK2 knockout mouse model does not fully recapitulate PD-like characteristics^42^. We randomized PARK2 knockout mice into rotenone (PARK2+R) or a vehicle regimen (PARK2+V) for 32 days to evaluate the effects of combining PARK2 knockout mutation with the chronic rotenone model (Fig. 7a). First, we evaluated if rotenone resulted in impairment of motor function in PARK2 mice. We found that PARK2+R mice had decreased latency to fall off (82.91 ± 5.97 SEM) and decreased rounds per minute (22.17 ± 1.54 SEM) on the rotarod tests compared to none-exposed mice (118 ± 11.38 SEM, *p* = 0.007 and 31 ± 2.5 SEM, *p* = 0.004, respectively) (Fig. 7b, c), which indicates that rotenone resulted in increased motor impairment. In addition, we evaluated the normalized muscle strength and found that PARK2+R had decreased muscle strength (6.11 ± 0.21 SEM) compared to non-exposed mice (6.91 ± 0.17 SEM, *p* = 0.01) (Fig. 7d). This indicates that rotenone resulted in decreased muscle function- and strength as described in Wt mice (Figs. 1 and 4). We did not observe any differences in the time to descend the pole tests (Fig. 7e). Based on the effects of rotenone exposure on motor behavior we speculated whether rotenone would result in exacerbation of non-motor impairment in PARK2 mice. We found that PARK2+R mice had significantly lower spontaneous alternation percentage in the Y-maze test (54 ± 35.4 range) compared to none-exposed mice (66.1 ± 3.95 SEM, *p* = 0.003) (Fig. 7g), which indicated that the cognitive impairment was further exacerbated by rotenone. Finally, we evaluated whether rotenone affected the serum glucose concentration in PARK2 mice and found that PARK2+R mice had hyperglycemia (8.26 ± 0.37 SEM) compared to PARK2+V mice (6.39 ± 0.93 SEM, *p* = 0.04) (Fig. 7h). Overall, the results indicate that rotenone exacerbates motor and non-motor features in the PARK2 knockout mice mimicking some aspects of PD symptoms^1^.

### Inhibition of CPT1 results in decreased non-motor impairment in PARK2 knockout mice exposed to rotenone

**Fig. 8:**
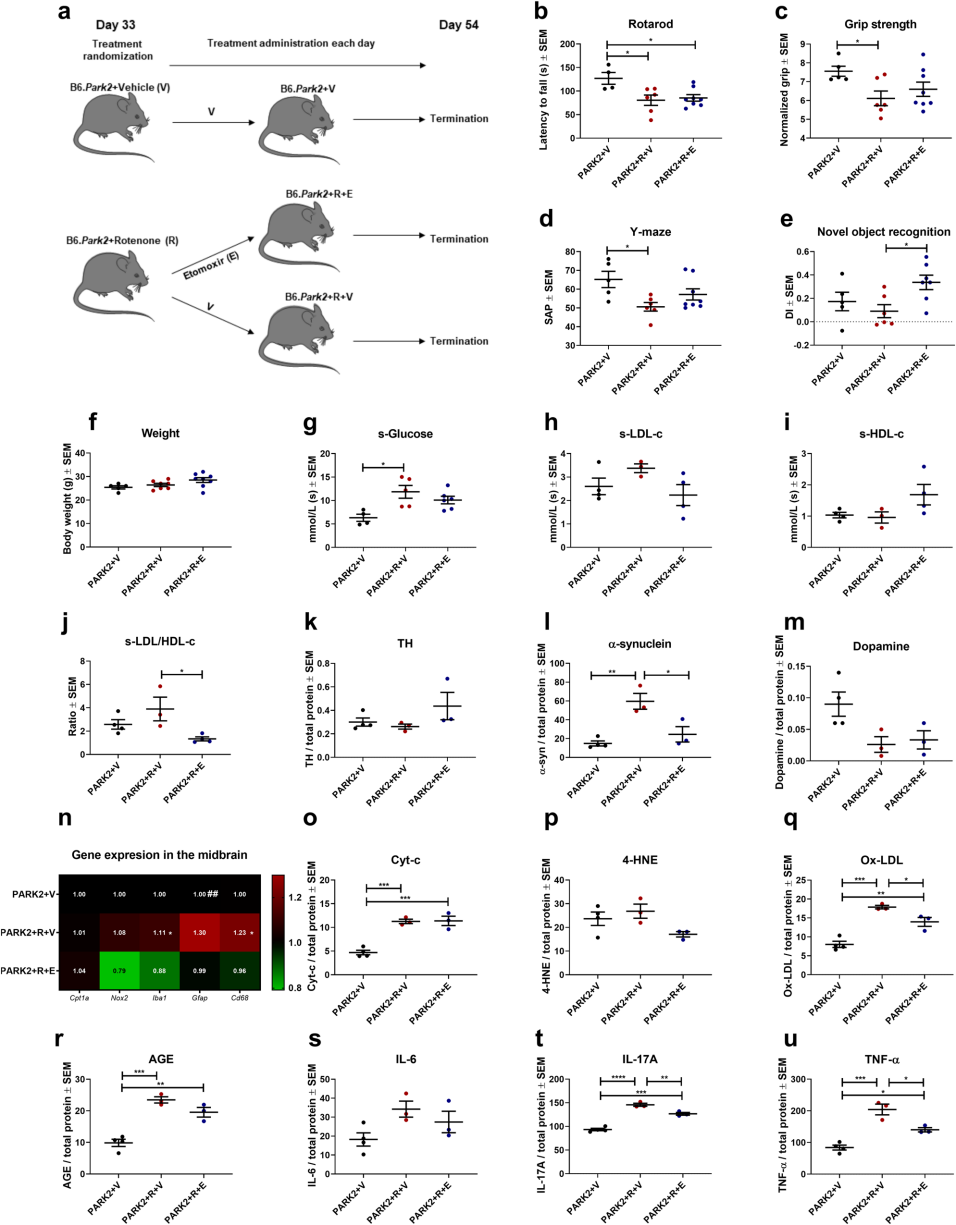
Downregulation of CPT1 activity by etomoxir results in amelioration of impaired object recognition, decreased LDL-c/HDL-c ratio and diminishes reactive microglia/macrophage gene expression in the midbrain in PARK2 knockout mice exposed to rotenone. **a** Male PARK2 knockout mice were randomized into etomoxir or vehicle treatment following rotenone (30 mg/kg) exposure for 32 days. **b** Mean latency to fall of the rotarod (*n* = 4–8). **c** Mean normalized grip strength (*n* = 5–8). **d** Visuospatial memory expressed as mean spontaneous alternation percentage in the Y-maze test (*n* = 5–8). **e** Mean discrimination index in the novel object recognition test (*n* = 5–7). **f** Mean weight of mice at day 54 in grams (*n* = 5–8). **g** Mean serum glucose levels measured in mmol/l (*n* = 4–6). **h** Mean serum LDL-c levels measured in mmol/l (*n* = 3–4). **i** Mean serum HDL-c levels measured in mmol/l (*n* = 3–4). **j** Mean ratio of serum LDL-c/HDL-c levels (*n* = 3–4). **k** TH levels in the midbrain expressed as mean ng/mg total protein (*n* = 3–4). **l** α-Synuclein levels in the midbrain expressed as mean pg/mg total protein (*n* = 3–4). **m** Dopamine levels in the midbrain expressed as mean ng/mg total protein (*n* = 3–4). **n** Heatmap illustrating mean normalized fold gene expression change in the midbrain of *Cpt1a, Cpt1c, Nox2, Pgc1a, Iba1, Gfap* and *Cd68* (*n* = 4–5). **o** Cytochrome-c levels in the midbrain expressed as ng/mg total protein (*n* = 3–4). **p** 4-Hydroxy-2-nonenal levels in the midbrain expressed as ng/mg total protein (*n* = 3–4). **q** Oxidized LDL levels in the midbrain expressed as ng/mg total protein (*n* = 3–4). **r** Advanced glycation end product levels in the midbrain expressed as ng/mg total protein (*n* = 3–4). **s** IL-6 levels in the midbrain expressed as pg/mg total protein (*n* = 3–4). **t** IL-17A levels in the midbrain expressed as pg/mg total protein (*n* = 3–4). **u** TNF-α levels in the midbrain expressed as pg/mg total protein (*n* = 3–4). Animals were tested at day 54 and serum and brain samples were obtained at day 54. Error bars represent the standard error of the mean (SEM). Data are representative of one experiment. Protein levels (TH, α-synuclein, and dopamine) were normalized to total protein concentration. RT-qPCR gene expression was normalized to *β-actin* and *Gapdh*. Significant differences for behavior, serum and protein experiments; *p ≤ 0.05; **p ≤ 0.01; ***p ≤ 0.001; ****p ≤ 0.0001. Significant differences in RT-qPCR experiments; ^#^significant difference between PARK2+V and PARK2+R+V, *significant difference between PARK2+R+V and PARK2+R+E. PARK2 PARK2 knockout genotype, R rotenone, V Vehicle, E etomoxir, SAP spontaneous alternation percentage, DI discrimination index, TH tyrosine hydroxylase. Center line = mean, and whiskers = standard error of the mean except for **g**, center line = median and whiskers = interquartile range. Statistics: one-way ANOVA followed by post hoc Tukey. We acknowledge Servier Medical Art for the mouse illustration, with the following license: https://creativecommons.org/licenses/by/3.0/. No changes were made to the drawing.

Rotenone resulted in more severe motor and non-motor impairment in PARK2 knockout mice. Thus, we speculated whether the downregulation of CPT1 activity by etomoxir could ameliorate the impaired motor and non-motor functions following the rotenone exposure. PARK2 mice were randomized into treatment with etomoxir (PARK2+R+E) or vehicle (PARK2+R+V) for a 21-day washout period, following rotenone exposure (Fig. 8a). After 21 days, the rotenone-exposed mice were evaluated for impaired motor and non-motor behavior. Both treatment groups of rotenone-exposed mice had a significantly lower latency to fall on the rotarod compared to non-exposed mice (Fig. 8b). In addition, PARK2+R+V mice had significantly lower muscle strength (6.11 ± 0.39 SEM, *p* = 0.05) compared to PARK2+V mice (7.56 ± 0.27 SEM), which was not the case for PARK2+R+E mice (6.6 ± 0.38 SEM, *p* = 0.2) (Fig. 8c). This indicated that etomoxir had some potential beneficial effect on muscle strength. However, we did not find any significant differences between the rotenone-exposed PARK2 mice, compared to rotenone-exposed PARK2 mice treated with etomoxir. This pointed toward that the rotenone-induced motor deficits combined with the PARK2 knockout resulted in too severe disease to be ameliorated by CPT1 inhibition. In addition, we also evaluated whether the downregulation of CPT1 activity resulted in the amelioration of non-motor impairments in the rotenone-exposed mice. PARK2+R+V mice had a significantly impaired visuospatial memory (50.61 ± 2.3 SEM) based on the SAP compared to PARK2+V mice (65.2± SEM, *p* = 0.02) (Fig. 8d). However, no difference in visuospatial memory between PARK2R+E (57.2 ± 2.99 SEM) mice and PARK2+V mice (*p* = 0.22) was identified. In conjunction with this, PARK2+R+E had a significantly higher DI (0.34 ± 0.06 SEM) compared to PARK2+R+V (0.09 ± 0.06 SEM, *p* = 0.04) (Fig. 8e). Indicating that downregulation of CPT1 following rotenone exposure could, to some degree, dampen the impaired recognition memory. Subsequently, changes in serum glucose, and lipoprotein concentrations were examined. We found that rotenone resulted in increased glucose concentrations but no difference between etomoxir and vehicle-treated mice was observed (Fig. 8g). PARK2+R+E had a significantly lower LDL-c/HDL-c ratio (1.34 ± 0.17 SEM) compared to PARK2+R+V (3.9 ± 1.01 SEM, *p* = 0.03) (Fig. 8h–j), which indicated that etomoxir, to some degree, decreased the pathological lipoprotein composition.

### Inhibition of CPT1 decreases α-synuclein concentration and attenuates normalized gene expression and inflammatory markers of reactive resident immune cells in the midbrain in PARK2 knockout mice exposed to rotenone

Next, we speculated whether the rotenone exposure resulted in the establishment of key PD-like biochemical changes in the PARK2 knockout mice. Therefore, we examined the normalized concentrations of TH, α-syn, and dopamine in the midbrain of these mice. We did not observe any significant differences in the TH protein concentrations between the groups (Fig. 8k). However, Rotenone exposure resulted in significantly higher normalized α-syn protein concentration in the midbrain (59.62 ± 8.45 SEM) compared to none-exposed (14.89 ± 2.65 SEM, *p* = 0.003) (Fig. 8l), which was not present in the PARK2+R+E (24.55 ± 8.19 SEM, *p* = 0.02), indicating that etomoxir potentially provides protection against α-syn deposition. As PARK2 mice are prone to DA neurodegeneration following complex I inhibition^45^, we hypothesized that rotenone would deplete dopamine in the midbrain. We found that following a 21-day washout period, dopamine levels were lower in rotenone-exposed PARK2 mice (0.03 ± 0.01 SEM) compared to non-exposed mice (0.09 ± 0.02 SEM) (Fig. 8m). However, the difference was not statistically significant (*p* = 0.07).

Furthermore, we evaluated whether the inhibition of CPT1 activity by etomoxir resulted in changes in normalized gene expression of metabolic, oxidative stress and reactive microglia/macrophages markers in the midbrain. PARK2+R+E mice had a significantly lower normalized expression of *Iba1* (0.88 ± 0.08 SEM) and *Cd68* (0.96 ± 0.07 SEM) compared to PARK2+R+V (1.11 ± 0.4 SEM *p* = 0.05, 1.23 ± 0.06 SEM, *p* = 0.04) (Fig. 8n), which indicates that etomoxir resulted in diminished levels of reactive microglia/macrophages.

Finally, we evaluated the normalized protein concentration of biomarkers of mitochondrial dysfunction, oxidative stress and inflammation in the midbrain. Rotenone resulted in significantly increased levels of Cyt-c (PARK2+R+V 11.26 ± 0.46 SEM, PARK2+R+E 11.37 ± 0.99 SEM) (Fig. 8o) compared to none-exposed (4.69 ± 0.47 SEM, *p* = 0.0004 and *p* = 0.0004, respectively), indicating mitochondrial dysfunction. We did not detect any difference in 4-HNE between the groups (Fig. 8p). However, rotenone resulted in increased levels of Ox-LDL (17.87 ± 0.45 SEM), which was significantly decreased by etomoxir treatment (13.96 ± 1.19 SEM, *p* = 0.05) (Fig. 8q). This could indicate decreased oxidative damage due to inhibition of CPT1. Rotenone resulted in significantly higher levels of AGEs in the midbrain (Fig. 8r), which was not affected by etomoxir. No significant difference was found in IL-6 levels in the midbrain but etomoxir resulted in significantly lower levels of IL-17A (126.7 ± 2.93 SEM) (Fig. 8t) and TNF-α (140.1 ± 6.83 SEM) compared to PARK2+R+V mice (145.7 ± 3.21 SEM, *p* = 0.007 and 204.2 ± 16.78 SEM, *p* = 0.01, respectively) (Fig. 8u). Overall, these results indicated that PARK2 knockout combined with a chronic rotenone exposure regimen resulted in irreversible damage but that inhibition of CPT1 had some effects on object cognition memory, LDL-c/HDL-c ratio, α-syn deposition, reactive microglia/macrophages and inflammation in the midbrain.

### Rotenone results in gut dysbiosis and this is reversed by the downregulation of CPT1

Over the last decade, PD has been shown to be a multisystem disease with pathology outside the CNS, e.g., α-syn has been located in the gut prior to the brain^51,52^. Multiple studies show that the gut microbiome is altered in PD patients and in vivo PD animal models^53–56^. The gut microbiome modulates metabolism and the systemic metabolism modulates the composition of the gut microbiome^25,32,57,58^. In this regard, CPT1A lipid metabolism modulates the fecal gut microbiome^32^ and etomoxir modulates the presence of specific bacteria^59^. In addition, the gut microbiome affects inflammation^54^ and disease mechanisms implicated in neurodegeneration^52,56^ by, e.g., leakage of inflammatory mediators through the intestinal barrier and communication through the enteric nervous system^24^. Based on this, and the fact that the gut modulates the metabolism and vice versa we evaluated the bacterial composition of the fecal gut microbiome in our PD mouse models (P479L rotenone experiment at day 32, Wt+R+E experiment at day 62, PARK2 vs Wt, PARK2+V vs PARK2+E, PARK2+V vs PARK2+R at day 32, and PARK2+R+E at day 54 experiment) using taxonomic marker gene sequencing (16S rRNA gene). First, we evaluated the bacterial community species α-diversity (defined as a difference in intragroup diversity^60^) and found that rotenone resulted in a non-significant increased α-diversity which was attenuated by inhibition of CPT1 (Supplementary Fig. 5). Next, we evaluated differences in β-diversity using principal coordinates analysis plots and found differences in β-diversity (defined as a difference in intergroup diversity^60^) between rotenone compared to non-rotenone-exposed groups, and mice with CPT1 inhibition compared to no inhibition (P479L+R, Wt+R+E, PARK2, PARK2+E and PARK2+R+E groups) (Supplementary Fig. 6). This indicated that the genotypes and treatments affected the gut microbiome and verse versa.

Based on this, we constructed heatmaps to illustrate differences in mean relative abundance of specific taxa at the phyla, class, order, and family and genus level (Supplementary Tables 1–6). Rotenone-exposed C57Bl/6J mice (Wt+R, exposed for 32 days) had a decreased relative abundance of *Verrucomicrobia* and an increase in *Cyanobacteria* at the phyla level (Supplementary Table 1). *Verrucomicrobia* communities inversely relate to fasting glucose, plasma triglycerides, and result in increased insulin sensitivity^61,62^. Interferon-ϒ-deficient mice have increased levels of *Verrucomicrobia* and are diminished in the Thy1 α-syn mouse model mimicking PD^63^. *Cyanobacteria* produce β-N-methylamino-L-alanine (BMAA) which is found in multiple neurodegenerative diseases^64,65^. In addition, BMAA administered to rats induce an amyotrophic lateral sclerosis (ALS)-like phenotype with profound inflammation, promote astrogliosis and trigger cytotoxic effects in multiple neuronal cell lines^66^. The result showed that gut microbiome composition was affected by rotenone exposure and may result in systemic decreased glucose metabolism due to insulin resistance and increased inflammation in the gut and thereby potential in the CNS. Differences at the other taxonomic levels were in accordance with the changes observed at phyla level (Supplementary Table 1). The Wt+R mice had a higher relative abundance of *Gastranaerophilales*, also observed in association with ALS, and colonic inflammation^67,68^. These mechanisms (insulin resistance, hyperglycemia, hyperlipidemia, increase in inflammatory cytokines and general cytotoxic effects) might explain some of the pathological effects seen in the chronic rotenone mouse model.

We then examined differences at the phyla level between Wt+R, and P479L+R mice (Supplementary Table 1). Wt+R mice had an increased relative abundance of *Cyanobacteria*, *Proteobacteria*, and decreased *Bacteroidetes*, *Verrucomicrobia*, and *Actinobacteria* compared to P479L+R mice. *Proteobacteria* are increased in PD, associated with metabolic syndrome, and intestinal inflammation^56,69^. *Actinobacteria* decreases in mice injected with LPS and correlates to depressive-like behavior^70^. We also examined differences at the other taxonomic levels, and found multiple differences, besides the ones observed at phyla level (Supplementary Table 1). Wt+R mice had an increased relative abundance of *Gastranaerophilales*, *Rikenellaceae*, *Desulfovibrionaceae*, *Rhodospirillales* and decreased abundance of *Prevotellaceae* compared to P479L+R mice. *Rikenellaceae* communities are increased in multiple CNS diseases, and are associated with a long duration of PD^56^. *Desulfovibrionaceae* are increased in PD^71^. *Rhodospirillales* are associated with diet-induced obesity^72,73^. *Burkholderiaceae* are increased in inflammatory bowel disease patients^56^. *Prevotellaceae* are decreased in PD patients with fast disease progression, and in general less abundant in patients with neurodegenerative diseases^56,74^. Thus, downregulating the activity of lipid metabolism, by CPT1A inhibition, resulted in increased levels of gut bacteria associated with protetive mechanisms and decreased levels of bacteria associated with pathological mechanisms.

**Fig. 9:**
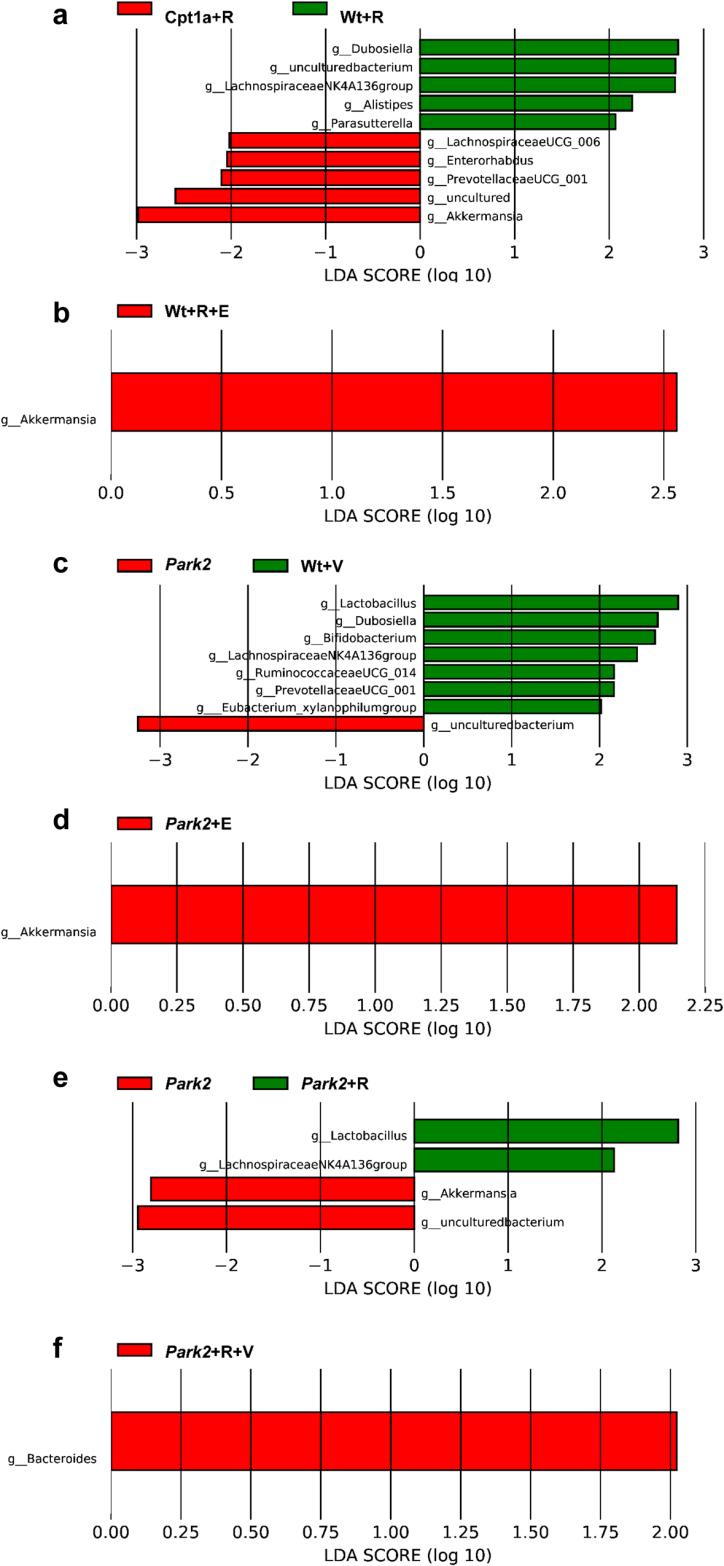
Linear discriminant analysis effect size (LEfSe) for organisms most like to explain differences in gut microbiome between treatment groups. **a** Organisms most like to explain differences between fecal gut microbiome between Wt+R and P479L+R (Cpt1a+R) mice at day 32 (Log10 fold change). **b** Organisms most like to explain differences between fecal gut microbiome between Wt+R+V and Wt+R+E at day 62 (Log10 fold change). **c** Organisms most like to explain differences between fecal gut microbiome between Wt and PARK2 (*Park2*) mice (Log10 fold change). **d** Organisms most like to explain differences between fecal gut microbiome between PARK2+V (*Park2*+V) and PARK2+E (*Park2*+E) mice at day 22 (Log10 fold change. **e** Organisms most like to explain differences between fecal gut microbiome between PARK2+V (*Park2*+V) and PARK2+R (*Park2*+R) mice (Log10 fold change) at day 32. **f** Organisms most like to explain differences between fecal gut microbiome between PARK2+R+V (*Park2*+R+V) and PARK2+R+E (*Park2*+R+E) mice at day 54 (Log10 fold change). Fecal samples were collected at the termination time point for 16s rRNA analyses targeting the V4–V5 region. *N* = 4–6. Data are representative of one 16s rRNA sequencing experiment. Wt Wildtype, Cpt1a+R *Cpt1a* P479L homozygote mice, *Park2* PARK2 knockout genotype, R rotenone, V Vehicle, E etomoxir. Statistics: LEfSe according to ref. ^75^.

In further detail, we used linear discriminant analysis effect size (LEfSe)^75^ to determine the organisms most likely to explain the differences in gut microbiome composition at the genus level between the sample groups (Fig. 9a). The observed changes to the gut microbiome (*dubosiella*, *akkermansia* and *alistepes*) might explain some of the significant disease differences observed for P479L+R compared to Wt+R mice (Figs. 1–3) possible due to mechanisms such as inflammation and disrupted glucose homeostasis. Conversely, this also indicated that the CPT1A P479L mutation had positive effects on the microbiome following rotenone exposure. Similarly, we have previously shown that P479L mice have changes in their fecal gut microbiome (decreased *Alistipes*, decreased *Akkermansia*, decreased *Rikenellaceae* and increased *Lachnospiraceae*, increased *Faecalibaculum*, and increased *Blautia*)^32^. The decreased communities were associated with mood disorders, volatile fatty acids, cortisol levels, type 2 diabetes, obesity and inflammation^32^. Whereas the increased communities were associated with factors such as diminished inflammation, attenuated gut permeability and decreased abundance in neurodegenerative diseases^32^.

We also evaluated gut microbiome differences between Wt+R+V and Wt+R+E mice at the phyla level. Wt+R+E had an increased relative abundance of *Verrucomicrobia* compared to the Wt+R+V mice (Supplementary Table 2). *Akkermansia* (*Verrucomicrobia*) was also the most likely organism to some degree to, potentially, explain the positive effects of the etomoxir treatment (Fig. 2) via increased glucose metabolism and decreased intestinal inflammation.

Motivated by the gut microbiome findings for the C57Bl/6J rotenone experiments, we examined differences in the fecal gut microbiome between Wt mice and PARK2 knockout mice using 16S rRNA abundance heatmaps (Supplementary Table 3) and LEfSe (Fig. 9c). Overall the differences were consistent with previous findings in humans with PD^56^. These included, but are not limited to, decreased *Lachnospiraceae*, *Rikenellaceae*, decreased *Lactobacillaceae*, decreased *Prevotellaceae*, and increased *Akkermansia*^56^. To the authors’ knowledge, this is the first time that changes in gut microbiome in PARK2 mice have been examined. We also examined whether PARK2 mice treated with etomoxir had any difference in their fecal gut microbiome compared to PARK2 mice treated with vehicle (Fig. 9d and Supplementary Table 4). Interestingly, etomoxir treatment also resulted in a significant increase in *Akkermansia* in PARK2 mice, consistent with the findings from P479L+R mice and the chronic Wt+R+E experiment.

PARK2 knockout mice exposed to rotenone had a significantly higher relative abundance of *Firmicutes*, *Cyanobacteria*, and significantly lower abundance of *Bacteroidetes*, and *Verrucomicrobia* compared to non-rotenone-exposed PARK2 mice (Supplementary Table 5). These findings were consistent with the changes seen in the rotenone-exposed C57Bl/6J mice (Supplementary Tables 1 and 2). The organisms at the genus level, which was most likely to explain the difference in the fecal gut microbiome, were identified as *Lactobacillus*, *Lachnospispiraceae*, and *Akkermansia* (Fig. 9e). Increased *lactobacillaceae* communities are associated with lower levels of ghrelin^56^, intellectual impairment, and more severe motor symptoms in PD patients^76^. Finally, we examined the effects of CPT1 inhibition fecal microbiome in the PARK2 rotenone-exposed mice using heatmaps (Supplementary Table 6) and LEfSe (Fig. 9f). We found that the difference between the groups was most likely driven by differences in *Bacteroidaceae* at the genus level. This indicated that overall, the inhibition of CPT1 by etomoxir had positive effects on the gut microbiome in the PARK2 mice.

Overall, analysis of the mice gut microbiome bacteria compositions supported that dysbiosis may play a role in the establishment and progression of disease in the chronic rotenone mouse models by mechanisms such as dysregulated glucose/lipid metabolism and inflammation.

## Discussion

This study investigates the effects of CPT1 downregulation on disease induction and progression in mouse models mimicking some aspects of PD. A single-point mutation (P479L) in CPT1A downregulates activity and protects against neurodegenerative diseases such as multiple sclerosis^29,30,77^, ALS^78^, and depression^79^, as observed in the Inuit population. Note, the genetical and pharmacological inhibition of CPT1A and CPT1 and not the CNS-specific CPT1C was due to the fact that CPT1C is located at the rough endoplasmic reticulum and does not participate in mitochondrial fatty acid oxidation^80^. Here we used PD mice models to see if there was a protective effect for this disease as well. Male mice were used in this study because male mice are more susceptible to rotenone-induced PD-like disease with higher levels of inflammation and deposition of α-syn compared to female mice, which is in accordance with the incidence in humans^81^. Nonetheless, it is important to consider the effects of sex in the evaluation of potential new drugs and targets. However, we have previously observed similar effects with CPT1 and CPT1A inhibition considering inflammatory- and oxidative stress levels in other animal models mimicking central nervous system diseases using female rodents^28–30,82^.

Mice harboring the P479L mutation (*Cpt1a*) expresses CPT1A with a 22% activity of CPT1A compared to Wt CPT1A^83^. After 32 days of rotenone administration, the Wt C57Bl/6J mice presented with pathological motor and non-motor behavior (Fig. 1c, d, g, j) as seen previously^31,55^. We found that rotenone treatment induced dysregulation of glucose and lipoprotein homeostasis (Fig. 2a, c)^12,13^, and importantly that CPT1A P479L mutant mice showed resistance to this pathology^32^ (Fig. 2). Evaluation of mice motor and non-motor behavior were based on surrogate markers for symptoms seen in PD patients^1^. In addition, in mice with PD induced through the administration of rotenone, we found that the CPT1 antagonist etomoxir was able to restore motor function, muscle strength^32^, and sensorimotor function^32^ during rotenone exposure (Fig. 1) and also non-motor behavior during a rotenone washout (Fig. 4d).

We also confirmed and supplemented novel markers of PD in symptomatic *Park2* knockout mice, such as dysregulated glucose, and lipoprotein homeostasis (Fig. 5j–n). Pathological motor and non-motor behavior were attenuated in PARK2 mice by the downregulation of CPT1 (Fig. 6c, e, f, h). However, we did not observe any amelioration of motor impairment in PARK2 knockout mice exposed to rotenone, although cognitive impairment was attenuated by etomoxir treatment (Fig. 8b–e). Several mechanisms may be responsible for the amelioration of pathological motor and non-motor behavior and resistance to disease induction as discussed below. PD was originally hypothesized to begin outside of the CNS and then directly target neurons^84^. However, recently a second hypothesis for the development of PD was proposed, known as the brain-first versus gut-first hypothesis^85^. Based on this hypothesis, in the gut-first (or peripheral nervous system first) hypothesis PD is presumed to begin outside the body and then spread to the CNS through the olfactory bulb or the gastrointestinal tract, whereas in the brain-first subtype form, PD is suspected to begin within the CNS and then spread to the rest of the body^85^. The brain-first subtype of PD is more associated with mutations in PD-associated genes^85^. This could also explain some of the differences observed in the Wt rotenone and the PARK2 mouse models.

Multiple pathophysiological mechanisms have been observed to play a role in PD, such as inflammation, dysregulation of metabolism, oxidative stress, insulin resistance and disruption of gut microbiome composition. Inflammation has been found to be a key mechanism in PD patients and animal models alike^86^. Rotenone blocks the electron transport chain complex I which in turn causes mitochondrial dysfunction via increased levels of Cyt-c, disruption of the mitochondrial membrane, and oxidative stress^38^. In addition, we have previously shown that genes associated with mitochondrial function and oxidative stress are altered in CPT1A P479L homozygote mutant mice^30^. Therefore, we evaluated whether the downregulation of CPT1 activity by the P479L mutation affected the levels of Cyt-c (marker of mitochondrial functional disruption) and metabolic markers of oxidative stress (4-HNE and Ox-LDL). We found that P479L mutant and Wt mice treated with etomoxir (Fig. 3) had decreased levels of Cyt-c and oxidative stress markers. As previously described, PARK2 mutant mice are characterized by increased oxidative stress (Fig. 5)^15,43^. Hence, we speculated whether the downregulation of CPT1 activity also affected oxidative stress in these mice (Fig. 6) or PARK2 mutant mice combined with rotenone (Fig. 8). It was found that the level of 4-HNE was significantly downregulated in PARK2 knockout mice (Fig. 6s) and ox-LDL in PARK2 mutant mice combined with rotenone exposure (Fig. 8q), following pharmacological CPT1 inhibition. This indicates that one of the mechanisms by which CPT1 downregulation ameliorates rotenone-induced motor and non-motor impairment could be by decreasing oxidative stress.

Recent studies based on PD patients and animal models have indicated that the metabolism of both glucose and lipids are dysregulated. High levels of glucose, diabetes mellitus type 2 and insulin resistance have been strongly associated with the disease^87–89^. Furthermore, the administration of insulin is protective against DA neuron death in a toxic rat model of PD^90^. Studies show that a shift from glucose to lipid metabolism occurs in PD and is clearly associated with insulin resistance^5–7,9,91^. Studies also show that downregulating CPT1 with etomoxir has a positive outcome on obesity and glucose metabolism^92,93^, Our study confirms that P479L mutant mice were resistant to rotenone-induced PD-mimicking disease, in part through a shift toward the metabolism of glucose over lipids. Rotenone increased glucose levels in C57Bl/6J mice, but not P479L+R mice (Fig. 2a), indicating differential effects. Interestingly, infants with CPT1A P479L mutation have an increased odds ratio for developing neonatal hypoglycemia^94^. CPT1A P479L mutations nor etomoxir do not cause complete inhibition of CPT1A mediated lipid metabolism but result in a shift toward glucose metabolism^92^. Rotenone has been shown to cause decreased expression of genes associated with glycolysis and increased expression of genes associated with fatty acid oxidation pointing toward differential effects^13^. Rotenone results in the inhibition of mitochondrial complex I, which can result in ATP depletion, oxidative stress, inflammation, and disruption of homeostasis^13^. Etomoxir was able to decrease glucose levels in C57Bl/6J, although not statistically significant in this data set (Figs. 2 and 4), and significantly in PARK2 mice (Fig. 6j). High glucose levels result in the production of AGEs, including glycation of proteins such as α-syn^39^. The glycated α-syn is prone to oligomerization and reduced protein degradation, resulting in the deposition of α-syn and Lewy body formation. This could potentially explain the lower level of α-syn in P479L mutated (Fig. 1) and etomoxir-treated mice (Figs. 3, 4 and 8), and could also be associated with the lower level of AGEs in the midbrain of these mice (Figs. 3 and 4). In support of this view, our results showed that the normalized protein concentration of TH was higher in mice with downregulated CPT1 activity (Fig. 3) which indicated that blocking β-oxidation affected DA neurons. Nonetheless, future stereological experiments will be necessary to confirm this. Remarkably, dopamine levels were high in P479L+V compared to Wt+V mice, whereas TH tended to be lower (Fig. 3). This could point toward a possible association between CPT1A activity and dopamine levels. However, the combination of PARK2 knockout induced oxidative stress^43^, disruption of glucose homeostasis^15^, and rotenone appeared too aggressive to be attenuated by etomoxir based on motor and non-motor behavior tests, TH, and dopamine levels in the midbrain (Fig. 8). Nevertheless, α-syn levels were significantly decreased (Fig. 8l) which may be associated with the changes in gene expression of reactive microglia/macrophage markers in the midbrain following etomoxir treatment (Fig. 8n).

Post-mortem studies, blood and CSF analysis, brain imaging and in vivo animal models have confirmed the presence of markers of inflammation such as reactive microglia expressing IL-6 and TNF-α^95^, inflammatory cytokines in CSF and serum^96^ and increased autoreactive T-cells^97^. Our research has indicated that blocking CPT1 using etomoxir is able to downregulate inflammation through modulation of both T- and B-cells^18,27–29,32^. We recently showed that *Cpt1a* mutant mice are resistant to immunologically induced EAE^30^. In addition, studies show that Ox-LDL increases inflammation^37^. Thus, we tested the downregulation of CPT1 and CPT1A in PD-mimicking mice and found that P479L+R (Fig. 3), Wt+R+E (Fig. 3), PARK2+R (Fig. 6) and PARK2+R+E (Fig. 8) mice had significantly lower expression of reactive microglia/macrophage markers (*Iba1*, *Cd68*) and decreased inflammation in the midbrain based on TNF-α levels (Figs. 3, 4, 6 and 8). Therefore, one of the mechanisms by which CPT1 inhibition, via P479L mutation or etomoxir, ameliorates rotenone-induced motor and non-motor impairment could be by reducing inflammation.

**Fig. 10:**
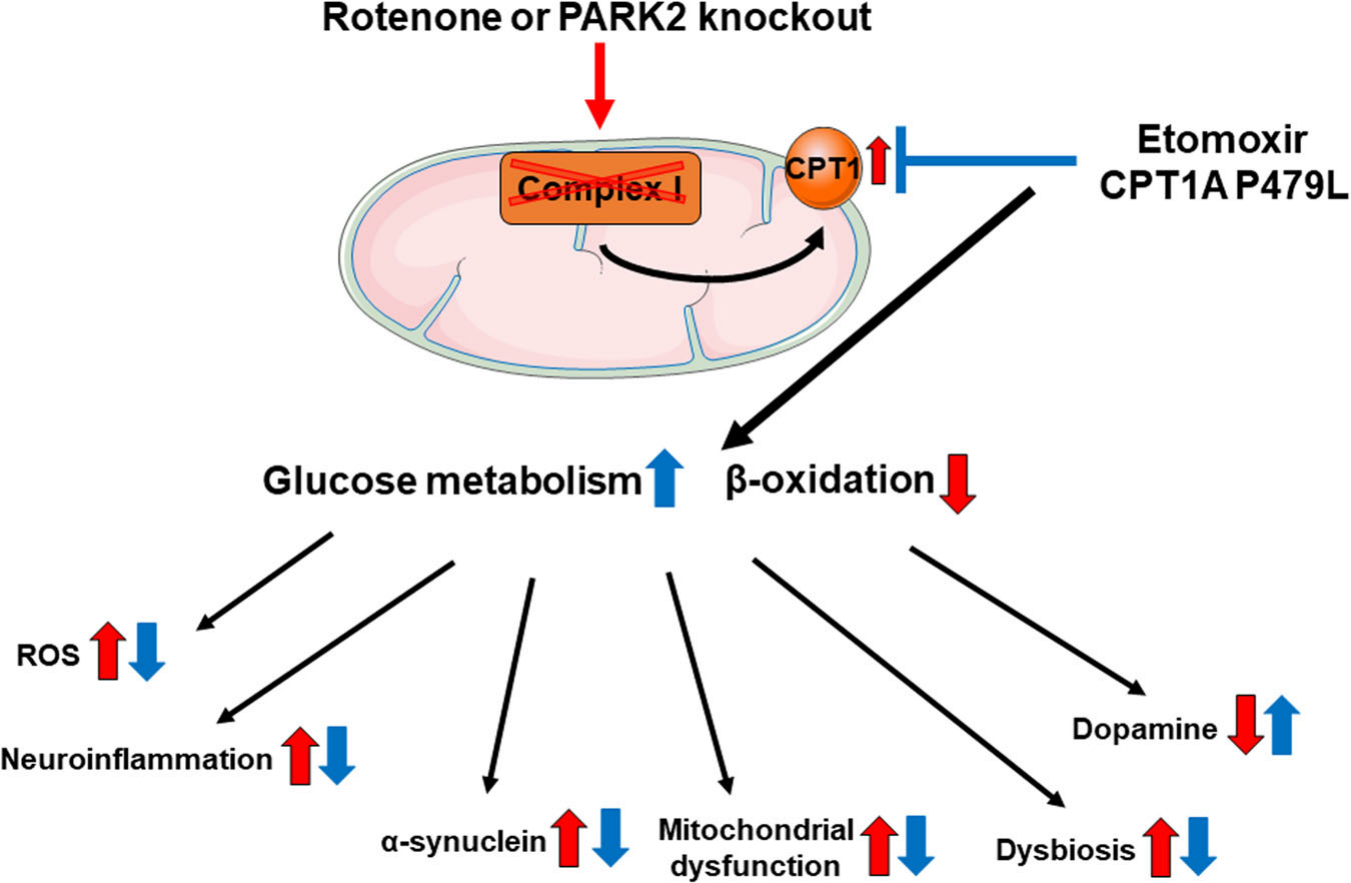
Inhibition of CPT1 counteracts rotenone-induced pathological mechanisms by targeting both extra- and intracerebral systems. Rotenone or PARK2 knockout results in blockade of mitochondrial complex I and upregulation of CPT1 and thereby β-oxidation. The upregulation of CPT1(A) is attenuated by the pharmacological antagonist etomoxir and the hypomorphic CPT1A allele (P479L mutation). Upregulation of CPT1 results in extracerebral effects such as the production of reactive oxidative species, production of pro-inflammatory cytokines, disruption of mitochondrial homeostasis and dysbiosis in the gut. Furthermore, this upregulation exacerbates intracerebral pathological mechanisms such as neuroinflammation, oxidative stress, aggregation of α-synuclein oligomers, mitochondrial dysfunction, which results in dopamine loss due to the death of dopaminergic neurons. Inhibition of CPT1(A) causes a shift toward glucose metabolism, causing amelioration of these disease mechanisms.

Interestingly, pharmacological inhibition of CPT1 and the CPT1A P479L mutation led to comparable effects (Figs. 1–3), despite differences in CPT1-isoform targeting (etomoxir has an affinity for all three isoforms). We argue, based on this and previous studies^28,30,32,79,82^, that this is because inhibition of CPT1 and CPT1A causes both extracerebral (e.g., diminished gut dysbiosis, increased leaked of inflammatory cytokines from the gut, decreased inflammation and oxidative stress in extracerebral organs such as muscles) and intracerebral effects (e.g., decreased inflammation, decreased oxidative stress, decreased blood–brain barrier disruption, decreased deposition of protein aggregates) (Fig. 10).

One of the etiological hypotheses regarding PD has been the involvement of the gut microbiome, specifically the β-oxidation, which plays a central metabolic role in the microbiota^25,54,57^. A study showed that oral gavage rotenone administration for 28 days induced changes in the microbiota, α-syn formation in the colon, motor impairment and loss of DA neurons in the substantia nigra^55^. Similarly, our results show that rotenone induces dysbiosis in the gut microbiome in C57Bl/6J mice (Fig. 9a, b, Supplementary Fig. 6a, b and Supplementary Tables 1 and 2) and, for the first time, in PARK2 knockout mice (decreased *Lachnospiraceae*, *Rikenellaceae*, decreased *Lactobacillaceae*, decreased *Prevotellaceae*, and increased *Akkermansia*) (Fig. 9c–f, Supplementary Fig. 6c–f and Supplementary Tables 3–6). These findings are in accordance with previous findings from humans with PD^56^. Here we also show that the administration of etomoxir can modulate the gut microbiome as previously indicated^57,98^. Inhibition of CPT1, leading to a downregulation of β-oxidation, resulted in a relatively greater abundance of bacterial communities associated with neuroprotective mechanisms, as described in the results section (Fig. 9). Interestingly, we found that a higher level of *Akkermansia* was present in P479L+R mice and Wt+R+E mice animals. Interestingly, the level of *Akkermansia* was lower in P479L+V compared to Wt+V mice, which could indicate differential effects following rotenone exposure. The effect of *Akkermansia* has been studied in obese and diabetic mice as well as type 2 diabetic patients. Here they showed that feeding the mice with a probiotic of *Akkermansia*, led to reversed effects of high-fat diet (HFD) and improved glucose tolerance. It was noted that in type 2 diabetes patients it was found that decreased levels of *Akkermasia* are associated with decreased insulin secretion^99,100^. Moreover, *Akkermansia* is involved in the gut-barrier integrity, where it was found that a decrease in mucus layer was associated with obesity, and that *Akkermansia* can restore the thickness of the mucus layer^99,100^. In addition, they found that Akkermansia is able to reduce inflammation^62,99^. These findings might explain what we observed in this study, as the glucose metabolism increases, and as the *Akkermansia* is able to “repair” the intestinal wall, we observed a decreased level of inflammatory markers within the CNS, possibly due to decreased leaked from the gut^24,52,54,56^. However, it has also been reported that increased or excess levels of *Akkermansia* in PD patients, where it is reported to be correlated with increased inflammation^101^. Therefore, to get a better understanding of *Akkermansia*’ role in PD models and PD patients, further analysis is needed to explore the role of gut mucus integrity and energy homeostasis. This is the first study to show that the downregulation of β-oxidation results in the alleviation of gut dysbiosis in PD mouse models. The changes in gut microbiome could potentially affect mechanisms such as gut permeability, inflammation, deposition of α-syn and thereby disease phenotype^54,56,82^.

The etiology of PD remains elusive; however, we hypothesized that the disease could be initiated by one or multiple stressors in genetically susceptible subjects (Supplementary Fig. 7). One such proposed mechanism is the hyperactivation of the HPA-axis and the production of glucocorticoids resulting in insulin resistance^21^. This insulin resistance results in high levels of glucose which in turn shifts the metabolism homeostasis toward the metabolism of lipids^87^. Accordingly, alleviating this shift in glucose versus lipid metabolism may be a therapeutic route for treating some forms of PD (Fig. 10).

In conclusion, this study shows that CPT1A P479L mutant mice are resistant to rotenone-induced PD, and that inhibition of CPT1 using etomoxir restores some aspects of motor and non-motor function, LDL-c/HDL-c ratio, TH levels, possibly decreases α-syn deposition and reduces gene expression of reactive microglia/macrophage markers in the midbrain in C57Bl/6J mice exposed to rotenone. Furthermore, we show that downregulation of the lipid metabolism (β-oxidation) via the CPT1 pathway alleviates pathological motor and non-motor behavior, disrupts glucose homeostasis and decreases gene expression markers of oxidative stress and reactive microglia/macrophages and astrocytes in PARK2 knockout mice. In addition, we show that PARK2 knockout combined with rotenone likely results in an augmented disease phenotype not attenuated by CPT1 downregulation following disease induction. Finally, we confirm that rotenone induces gut dysbiosis in C57Bl/6J consistent with previous studies, and for the first time in PARK2 knockout mice. We show that this dysbiosis is alleviated by the downregulation of CPT1 by etomoxir or by CPT1A P479L mutation in chronic rotenone-exposed mice, possibly by the shift toward more decreased gut permeability and anti-inflammatory compositions. Moreover, these data indicate that CPT1, and in particular CPT1A, plays a role in the chronic rotenone mouse model and PARK2 knockout model mimicking some aspects of PD. However, more mechanistic studies assessing changes in, e.g., inflammatory cytokines, mitochondrial biogenesis and oxidative damage within and outside the central nervous system are needed.

## Methods

### Animals

All animal experiments within this study were based on the ARRIVE guidelines. The experimental procedures were approved by The Danish Animal Experiment Inspectorate (2017-15-0201-01328). Animal welfare was assessed daily. Weight was assessed twice a week and if the weight was decreased by 20%, it was a direct criterion for early termination. The experimental operators were blinded to intervention, and treatment of the animals. Samples sizes were based on similar studies in the literature^31,55^, and were estimated using Gpower.

#### Mouse strains

C57BL/6J mice were obtained from Janvier Labs (Le Genest-Saint-Isle, France). Mice were acclimated for three weeks at 21 °C in a high-barrier animal facility. Male B6.129S4-Prkntm1Shn/J^42^ (JAX stock #006582) (*Park2*) mice were purchased from Jackson Laboratory (Bar Harbor, Maine, USA). The *Park2* colony was maintained by breeding homozygote *Park2* female together with *Park2* male mice at the animal facility (Aalborg, Denmark). Only *Park2* males were used for subsequent experiments. Mice were housed in groups of up to five in individually ventilated cages with a 12-h light-dark cycle, and ad libitum access to food and water. *Cpt1a* P479L (*Cpt1a*) mice were generated in collaboration with Netherlands Cancer Institute (Mouse Clinic for Cancer and Aging transgenic facility, Amsterdam, MGI-number 5810634) as previously published^30^. The colony was maintained at Aalborg University by breeding homozygote males, and females together. Only males were used for subsequent experiments.

### Chronic rotenone mouse model

Rotenone is a broad-spectrum insecticide, which blocks complex one in the mitochondria, and has been used in a variety of animal models mimicking neurodegeneration, and PD^31,55,102^. We used chronic oral gavage dosing^31,55^. Mice were randomized into groups exposed to 30 mg/kg rotenone (Sigma, CAS: 83-79-4) suspended in 0.5% Carboxymethylcellulose sodium salt (CMC) (Sigma #9004-32-4) or a group receiving CMC as vehicle daily for 32 days. At day 32, animals, who did not show signs of disease, were defined by the presence of a minimum two of the following factors: lower grip strength, lower latency to fall on the rotarod, longer time to transverse the beam, pole or lower number of rears in the cylinder test compared to the healthy control group was excluded from treatment groups (*n* = 6).

### *Cpt1a* P479L rotenone exposure experiment

C57Bl/6J, and *Cpt1a* P479L mice were exposed to rotenone (C57Bl/6J, *n* = 18; *Cpt1a*, *n* = 11) or CMC/vehicle (C57Bl/6J, *n* = 10; *Cpt1a*, *n* = 12) for 32 days. At day 32, mice were evaluated for pathological motor and non-motor behavior, as described below. A minor part of the behavior data, obtained from a pilot study without *Cpt1a*+CMC has been published previously by the authors^32^. Not all mice were evaluated by all tests. Fresh fecal samples were collected and immediately stored at –80 °C until further processing. Subsequently, mice were euthanized by cervical dislocation, blood was obtained by retroorbital puncture, and brains were quickly dissected and placed at –80 °C until further analysis. Blood was placed at room temperature for 30 min and centrifuged at 4500 RPM for 15 min. Following, the centrifugation the supernatant was harvested and stored at –80 °C until further analysis. All blood draws were obtained in the morning for all the groups.

### C57Bl/6J rotenone etomoxir experiment

At day 33, 12 weeks old C57BL/6J male mice exposed to rotenone (*n* = 18) were randomized into two groups. One was receiving etomoxir at 5 mg/kg dissolved in olive oil, and another received olive oil as vehicle. At this point, the daily administration of rotenone was changed to every second day; thus, the next rotenone administration was day 34, 36, etc. Furthermore, at day 33, the etomoxir intervention group was administered 5 mg/kg etomoxir suspended in olive oil and the vehicle group was administered equal amounts of vehicle (olive oil). This intervention was given every second day in between the rotenone and CMC administration, day 33, 35, etc. At day 62, the experiment ended. Thus, making it a total of 43 Rotenone administrations spanning 61 days. The etomoxir intervention group had 18 administrations of Etomoxir over a span of 28 days, receiving etomoxir without rotenone intervention from day 50 until 56 and again from day 58 until 60. A group of 12 weeks old C57BL/6J male mice (*n* = 5) was not exposed to rotenone and received CMC/vehicle during the treatment period. A minor part of the behavior data, obtained from this study, has been published previously^32^. However, this data is not presented in this paper. Mice were evaluated for pathological motor behavior using rotarod, grip strength, cylinder test, and beam test as described below. The acute rotenone experiment was performed before the implementation of the cognitive behavior tests. Therefore, non-motor behavior was not evaluated in this experiment. Fresh fecal samples were collected, and immediately stored at –80 °C until further processing at day 62. Following the collection of fecal samples, mice were euthanized by cervical dislocation, and blood samples were obtained as described above. Following blood sampling, brains were rapidly dissected, put on ice and stored at –80 °C until further analyses. All blood draws were obtained in the morning for all the groups.

### C57Bl/6J rotenone washout etomoxir experiment

At day 33, 15 weeks old C57BL/6J male mice exposed to rotenone (*n* = 18) were randomized into two groups. One was receiving etomoxir at 5 mg/kg dissolved in olive oil, and another received olive oil as vehicle. At this point, the exposure to rotenone was terminated. Etomoxir/vehicle was administered each day by oral gavage until day 54. Thus, making it a total of 21 treatment days. A group of 15 weeks old C57BL/6J male mice (*n* = 10) was not exposed to rotenone and received vehicle during the treatment period. A final group of 15 weeks old C57BL/6J mice (*n* = 5) was not exposed to rotenone, and received etomoxir, 5 mg/kg dissolved in olive oil, during the treatment period. Mice were evaluated for pathological motor and non-motor behavior at day 54 using grip strength, Y-maze, and dark-light box test, as described below. Fresh fecal samples were collected and immediately stored at –80 °C until further processing at day 54. Following the collection of fecal samples, mice were euthanized by cervical dislocation, and blood samples were obtained as described above. Following, brains were rapidly dissected and stored at –80 °C until further analyses. All blood draws were obtained in the morning for all the groups.

### *Park2* etomoxir experiment

Twenty-four weeks old *Park2* male mice were randomized into treatment with etomoxir (*n* = 7) or vehicle (*n* = 6) for 22 days. At day 22, mice were evaluated for pathological motor and non-motor behavior using rotarod, grip strength, Y-maze, novel object recognition, and dark-light box test, as described below. Fresh fecal samples were collected and immediately stored at –80 °C until further processing ay day 22. Mice were euthanized by cervical dislocation, and blood samples were obtained as described above. Next, brains were rapidly dissected and stored at –80 °C until further analyses. All blood draws were obtained in the morning for all the groups.

### *Park2* rotenone washout etomoxir experiment

At day 33, 24 weeks old *Park2* male mice exposed to rotenone (*n* = 13) were randomized into two treatment groups. One was receiving etomoxir at 5 mg/kg dissolved in olive oil (*n* = 8), and another received olive oil as a vehicle (*n* = 6). Rotenone exposure terminated at day 33. A group of *Park2* male mice (*n* = 5) was not exposed to rotenone and received vehicle during the treatment period. At day 54, mice were evaluated for pathological motor and non-motor behavior using rotarod, grip strength, Y-maze, and novel object recognition, as described below. Fresh fecal samples were collected and immediately stored at –80 °C until further processing. Following the collection of fecal samples, mice were euthanized by cervical dislocation, except two, blood samples were obtained as described above. Following, brains were rapidly dissected and stored at –80 °C until further analyses. Two mice from each treatment group were anesthetized using Isoflurane and intracardiac perfused with 1x PBS followed by 4% PFA, brains were collected, and placed for storage in 4% PFA/PBS at 4 °C. for 24 h and transferred to 70% ethanol^29^. All blood draws were obtained in the morning for all the groups.

### Behavioral tests

During the study, the mice underwent several state-of-the-art behavioral tests, which evaluated their abilities regarding motor, sensory-motor functions, anxiety-like behavior, and Visuospatial memory. Between every test, a full cleaning protocol by 70% ethanol was applied to the surfaces of the equipment. The mice were exposed to behavioral tests in a randomized order to avoid biases. All behavioral tests except rotarod and grip strength test were video recorded to avoid noise and freezing behavior by the animals. The experimenters were blinded to treatment groups for all behavioral studies, and mice were acclimatized to the laboratory 30 min before tests began. All tests were performed between 10:00 and 16:00 in the lights-on cycle. Video material was rated by three to four blinded individuals, depending on the experiment, which was instructed prior to rating. Rating was done based on predetermined rating protocols. Animals were excluded from behavioral tests if the video camera stopped recording before the test was done. Furthermore, animals were excluded from behavioral tests if they did not comply with the predetermined protocols as described under the different tests. The behavioral test battery was developed during the different experiments. The C57Bl/6J+rotenone with alternating treatment was conducted before the Y-maze, dark-light box test and novel object recognition tests were available and therefore none of the non-motor tests were applied in this study. The test battery was developed and optimized during the experiments, and therefore not all the sub-experiments included all the behavioral tests.

#### Rotarod performance test

Motor function was assessed using a rotarod (Rotamex-5 Rota Rod, Columbus Instruments, Columbus, Ohio, USA)^103^. Mice were placed on the accelerating rotarod, and the time the animals remained on the rotarod, and the maximum speed (RPM) was recorded. The speed was increased from 5 to 40 RPM within 140 s for the C57Bl/6J rotenone etomoxir experiment^32^. The speed was optimized for the other experiments, using an acceleration of 2.5 RPM per 10 s. Starting at 5 RPM and ending at 40 RPM at 140 s. The cut-off time was set to 180 s. Each animal received three trials per session. Prior to the rotarod performance test, mice were trained on three different dates with three trials per session. Rotarod test data are presented as mean latency to fall ± SEM (s) and mean RPM ± SEM. Data were excluded if the mice continuously jumped off the rotarod or turned in the wrong direction before acceleration as described in the protocol.

#### Grip strength

Muscle strength assessment was measured using a grip strength apparatus (BIOSEB, model: BIO-GS3, Chaville, France) gently pulling the animal by the tail^104^. Each animal received five trials. Data were excluded when the animal was making movements during the procedure or having an insufficient grip. Mean grip strength was calculated for each animal, and normalized to its body weight^104^. Grip strength data are presented as mean normalized grip strength (g/g) ± SEM. Data were excluded if the mice were unable to grasp the grid due to no interest according to the protocol.

#### Cylinder test

Spontaneous activity was assessed using a transparent cylinder in a low-illuminated, quiet room^105^. The mouse was placed in the cylinder and the total number of times that the mouse would stand on its hind limbs with its forelimbs against the cylinder wall (rears) was counted. The test was recorded by a video camera and rated by three blinded raters. The test lasted 3 min, and each animal received one session. Data are presented as mean number of rears ± SEM. Data were excluded if the mice jumped out of the cylinder before the trial ended according to the protocol.

#### Challenging transversal beam test

The challenging transversal beam test has been used in several animal models to assess sensorimotor function^105,106^. The test was conducted according to the protocol^105^. Mice had to traverse two different beams; the first being with a width of 16 mm at the broadest and narrowing down to 12 mm, and the second with a width of 6 mm. A video camera was used to record each trial. The total traverse time was noted. Prior to the challenging transversal beam test, the mice were trained until they were able to spontaneously cross the beam^105^. Data are presented as mean total time to transverse (s) ± SEM. Data were excluded if the mice turned around continuously or walked in the wrong direction as described in the protocol.

#### Pole test

The pole test was conducted to evaluate motor coordination^55^. The pole was constructed of wood measuring 50 cm long and 1 cm in diameter, wrapped by firmly glued metal mesh to facilitate grip preventing the mouse from sliding down. Briefly, a mouse was placed head up near the top of the pole and the times to turn the head down and descend the pole were measured. All mice were habituated to the test on two consecutive days with three trials per day. Each animal received three trials, which was recorded and rated by three blinded raters. Data are expressed as mean time to descend (s) ± SEM.

#### Y-maze

The Y-maze test is used to evaluate visuospatial memory (SAP) in rodents, and was done as previously described^107^. Y-maze was constructed according to MazeEngineers (USA) description. Mice were placed in the maze for 5 min and recorded by video. Following, the video was evaluated by three blinded raters and the total number of alternations and the total number of entries was recorded and based on this the spontaneous alternation percentage was calculated. Data are presented as mean number of entries ± SEM, and mean SAP ± SEM. Data were excluded if the mice did not conduct a minimum of predefined number of entries according to the protocol.

#### Dark-light box test

Anxiety-like behavior was evaluated by use of the dark-light box tests. The test is based on an innate tendency of mice to divert from illuminated areas as well as their spontaneous exploratory behavioral nature. An anxiety-like behavior is interpreted when mice tend to spend more time in dark areas. The dark-light box was performed in a box with two compartments (MazeEngineers, USA). The illuminated compartment was made of transparent materials which allows light to illuminate the area. The other compartment was completely dark since the materials including the lid of the construction breaks all light. The mouse was placed in the center of the illuminated area at the test start and the test was conducted for 5 min^108^. The test was recorded by a video camera and subsequently analyzed by three blinded raters. Two parameters were evaluated, the time spend in the light area versus the dark area, and were counted in seconds, and calculated as a ratio. A higher ratio is interpreted as a more anxiety-like behavior. The other test parameter was time in seconds from the start of the test until the mouse enters the dark compartment for the first time. A faster entry to the dark compartment is interpreted as anxiety-like behavior. Data are presented as ratios between compartments, and the time to enter the dark compartment (s). Unfortunately, video material for some of the animals was incomplete and could not be reviewed by the blinded raters.

#### Novel object recognition test

Spatial memory assessment was evaluated by use of the novel object recognition test^107,109^. The test uses the spontaneous exploratory behavior and curios nature of mice to differentiate between previously known objects and novel objects. If spatial memory is intact, then the mice will tend to investigate the novel object more than the familiar object. The test was performed in a rectangular open field box. First, the mouse was placed in the box for habituation for 10 min and then removed. Second, two identical objects were placed at every end of the box and then the mouse was reintroduced for 10 min. Finally, one of the objects was switched with a novel object of different size, and shape in 10 min, and the session was recorded^110^. Data were quantified as seconds of time spent in the marked areas around the objects. Time was noted when any part of the mouse was in contact with the marked area. Data are presented as a DI which allows discriminating between novel (TN) and familiar (TF) exploration time. Results are presented as a ratio between +1 and –1. A positive score indicates a tendency to explore the novel object. A ratio of 0 indicates no preference for any of the objects. The novel object recognition test was recorded by video camera and rated later. Unfortunately, video material for some of the animals was incomplete and could not be reviewed by the blinded raters.

### Serum glucose assay

Serum glucose levels were assessed using a colorimetric glucose assay (Crystal Chem, #81692) according to the manufacturer’s protocol. Standards and samples were assessed in duplicates. The assays were analyzed using a multiplate reader (PerkinElmer, Enspire). The concentration was calculated according to the standard curve method, and expressed as mmol/l ± SEM.

### Serum low-density lipoprotein-cholesterol assay (LDL)

Serum LDL levels were assessed using a colorimetric LDL assay (Crystal Chem, #79980) according to the manufacturer’s protocol. Standards and samples were assessed in duplicates. The assay was analyzed using a multiplate reader (PerkinElmer, Enspire). The concentration was calculated according to the standard curve method and expressed as mmol/l ± SEM.

### Serum high-density lipoprotein-cholesterol (HDL) assay

Serum HDL levels were assessed using a colometric HDL assay (Crystal Chem, #79990) according to protocol. Standards and samples were assessed in duplicates. The assay was analyzed using a multiplate reader (PerkinElmer, Enspire). The concentration was calculated according to the standard curve method and expressed as mmol/l ± SEM.

### Protein isolation

Collected brains were stored frozen in –80 °C. For protein isolation brains were taken out from storage and placed in the chilled mouse brain matrix (Zivic Instruments, #BSMAS001-1*)* and cut into 1 mm coronal sections. Striatum was isolated from two forebrain sections from both hemispheres under the stereotactic microscope, placed in the dissection buffer (Sucrose 300 mM, Imidazole 25 mM, EDTA 1 mM, pH 7.2 with the addition of proteinase inhibitors 0.4 μg/ml leupeptin, 0.1 mg/ml pefabloc) and homogenized with for 15 s. Resulted homogenate was centrifuged 4000 g/15 min at 4 °C and the amount of proteins was assessed with BCA kit (Thermo Fisher Scientific, #23225) in supernatant containing cytosolic fraction.

### SDS-page, and semiquantitative immunoblotting

Amount of proteins was equalized among samples and disulfide bonds were reduced by the addition of Laemmli sample buffer and heating to 95 °C for 5 min prior to loading on any-kD polyacrylamide gels (Bio-Rad, 4561086). Proteins were transferred with Bio-Rad system on methanol activated PVDF (polyvinylidene fluoride) membrane (0.45 μm pore size, Millipore) and unspecific antibody binding was blocked by incubation in 5% skimmed milk for 1 h at RT. Furthermore, membranes were washed with PBS-T and incubated overnight at 4 °C with primary antibodies for TH at dilution of 1:1000 (Thermo Fisher Scientific, #OPA1-04050) and the sites of antibody–antigen reaction were visualized with horseradish peroxidase-conjugated secondary antibodies (P448, diluted 1:3000; DAKO, Glostrup, Denmark) followed with chemiluminescence kit detection (ECL), and bands chemiluminescence level was read with Li-Cor (Li-Cor Odysses Fc). Band density was quantified by ImageJ (NIH Image, NIMH, NIH, Bethesda, MD, USA). For this, the band intensity from each lane was subtracted from the background intensity of that film. The labeling density values of a given protein were corrected by densitometry of corresponded β-actin and were normalized to facilitate comparisons. The measured band densities were pooled and expressed as the fraction of the control level (Wt+V group).

### Enzyme-linked immunosorbent assays (ELISAs)

The midbrain was isolated from the brains obtained from the respective animal experiments according to previously published method^111^. Tissues were homogenized in 1 ml ice-cold 1× PBS using a mechanical homogenizer. Afterward, tissue samples were centrifuged at 5000 RPM for 5 min. The supernatant was transferred to clean Eppendorf tubes, and immediately used for subsequent ELISAs. Standards, and samples were run in duplicates. TH levels were determined using a commercial sandwich ELISA (Aviva Systems Biology, #OKEH07185) according to the manufacturer’s protocol. α-Syn levels were analyzed using a commercial, sandwich ELISA (Biosite, #EXX-IR6NL6-96) according to the manufacturer’s protocol. Dopamine levels were assessed using a commercial, competitive ELISA (Biosite, #EXX-MGG9K6-96) according to the manufacturer’s protocol. Cyt-c (Biosite, #EKX-UJ5PRQ-96), 4-HNE (Biosite, #EKX-JGAVG3-96), Oxidized LDL (Biosite, #EKX-0Y6J7B-96), Advanced glycation end products (Biosite, EKX-BHU8AA-96), IL-6 ELISA MAX (BioLegend, #431301), IL-17A ELISA MAX (BioLegend, #432504) and TNF-α ELISA MAX (BioLegend, #430901) levels were assessed using commercial ELISA kits according to the manufacturer’s protocols. All assays were read at 450 nm using a multiplate reader (PerkinElmer, Enspire). Concentrations were interpolated using a standard curve method by applying a Sigmodial 4PLx fit. Levels of proteins were expressed as protein/total protein ± SEM. Total protein level in samples was determined using NanoDrop (Thermo).

### Reverse transcriptase quantitative polymerase chain reaction (RT-qPCR)

The midbrain was quickly isolated from brains from the respective treatment groups according to protocol^111^. RNA was extracted using a GeneJET RNA Purification Kit (K0731, Thermo Fisher) followed by cDNA synthesis using a quantity of 200 ng RNA by Maxima First Strand cDNA Synthesis Kit (K1671, Thermo Fisher)^30^. RT-qPCR was performed using Maxima SYBR Green qPCR Master mix (Thermo Fisher) with the following program: 1×: 95 °C for 10 min and 40×: 95 °C for 30 s, 60 °C for 30 s, and 72 °C for 30 s^30^. Primers were ordered from TagCopenhagen (Denmark) (Table 1), and the following primers were purchased from Qiagen (Germany): *Th* (# PPM05014A), *Cd68* (# PPM03976A), *Mbp* (#PPM04745F). Ct-values in Fig. 3 were determined in triplicates, and in duplicates for Figs. 4–6 and 8. The gene expression of the gene of interest was normalized to *β-actin*, and *Gapdh*. Data were normalized to respective control groups, and expressed as mean fold gene expression change ± SEM.

**Table 1:** Primer sequences for primers from TagCopenhagen.

Primer	Forward	Reverse
*β-actin*	CTGTCGAGTCGCGTCCACC	TCGTCATCCATGGCGAACTGG
*Gapdh*	GTG GACCTCATGGCCTACAT	TGTGAGGGAGATGCTCAGTG
*Cpt1a*	TCATCAGCAACCGGCCCAAA	GGAGGTTGTCCACGAGCCAG
*Cpt1c*	CTGACCTCTGACCGGTGGGC	TTTTCCAGGAGCGCAGGG
*Nox2*	TGGACGGCCCAACTGGGATA	TTCAGCCAAGGCTTCAGGGC
*Nrf2*	CGCCAGCTACTCCCAGGTTG	GGGGATATCCAGGGCAAGCG
*Pgc1α*	CACCGCAATTCTCCCTTGTA	TGCGGTATTCATCCCTCTTG
*Iba1*	GTTCCCAAGACCCATCTAGAGCTG	AGTTGGCTTCTGGTGTTCTTTGTTT
*Gfap*	ACAGACTTTCTCCAACCTCCA	CAGGGCTCCATTTTCAATC

### 16s rRNA sequencing on fecal pellets

DNA from fecal pellets was extracted using a *Quick*-DNA Fecal/Soil Microbe DNA Miniprep Kit (Zymo Research, #D6010) according to the manufacturer’s instructions. Afterward, samples were handed over to DNAsense ApS (Aalborg, Denmark) for library preparation, sequencing, and bioinformatics processing^32^. β-diversity was visualized using principal coordinate analysis. LEfSe^75^ was used to determine the features (organisms) most likely to explain differences between all combinations of classes (sample groups). The analysis was applied with default settings to OTU tables restricted to the genus level.

### Applied statistics and reproducibility

All data were analyzed using GraphPad Prism 8.0 software. Equality of variance was determined using *F*-test, or Brown–Forsythe test. If equality of variance was not met, Welch correction or Brown–Forsythe ANOVA test was carried out. Normality was assessed using the Shapiro–Wilkinson test, and Q/Q-plot. If the criteria of normality were met, a two-tailed unpaired *t*-test, one-way ANOVA or two-way ANOVA was conducted followed by the Tukey post hoc test. If the criteria of normality were violated, non-parametric Mann–Whitney *U* test or Kruskal–Wallis test followed by Dunn post hoc test was performed. If data were normally distributed, data were presented as mean ± SEM, and otherwise median ± interquartile range. Assessments with *p* < 0.05 were considered significant. Significance level were denoted by *p ≤ 0.05; **p ≤ 0.01; ***p ≤ 0.001; ****p ≤ 0.0001. Outliers were evaluated using the Grubbs test, and if present, removed. *Cpt1a* mutant animal experiments were repeated twice and produced similar results. All other experiments were performed once.

### Reporting summary

Further information on research design is available in the Nature Research Reporting Summary linked to this article.

## Supplementary information

Supplementary Material

Reporting Summary

## Data Availability

Data are available upon request to the corresponding author. The 16S sequencing data are available at NCBI using accession number BioProject ID PRJNA895962.
