# Supplementary material for: Inhibition of carnitine palmitoyl-transferase 1 is a potential target in a mouse model of Parkinson’s disease
Source: NPJ Parkinsons Dis. 2023 Jan 21;9:6. doi: 10.1038/s41531-023-00450-y (PMC9867753; doi:10.1038/s41531-023-00450-y)
Supplement: Supplementary file 1 — Supplementary Material [file 41531_2023_450_MOESM1_ESM.pdf]

Supplementary Figures and Tables

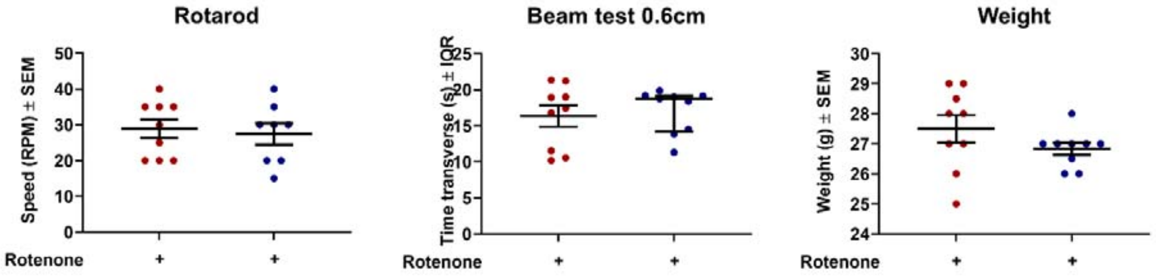

Supplementary Figure 1: Baseline following rotenone exposure for 32 days in the chronic rotenone study before treatment start

Wt mice were exposed to rotenone by oral gavage and tested at day 32 before randomization into treatment groups (group 1 and group 2, n= 9). No significant differences were observed indicating proper randomization. Wt: wild type. Statistics: Two-tailed unpaired t-test or Mann-Whitney U test.

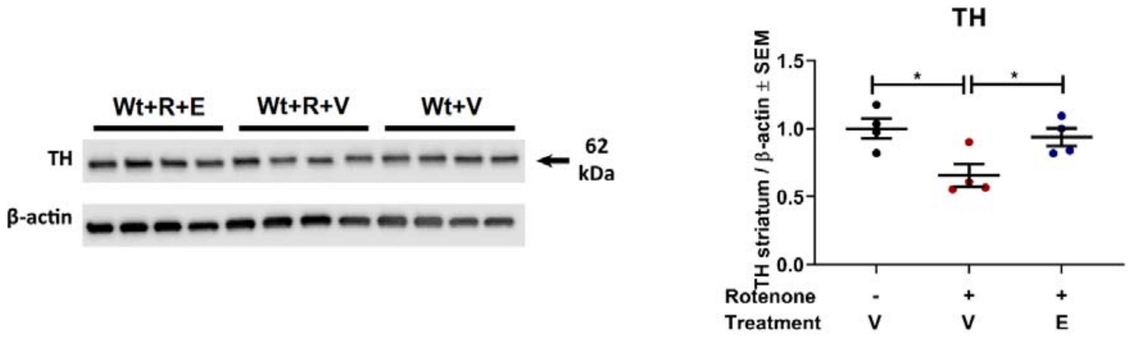

Supplementary Figure 2: Western blot and densitometry for TH protein level in the striatum in mice from the chronic rotenone exposure study

TH western blotting in the striatum with β-actin as reference protein (n=4) and densitometry of TH western blot in striatum illustrating mean TH-levels referenced to β-actin normalized to TH/ β-actin ratio in Wt+V mice (n=4). Significant differences: *$p \leq 0.05$. Wt: wild-type, R: rotenone, V: Vehicle, E: etomoxir, TH: Tyrosine hydroxylase. Statistics: One-way ANOVA followed by Tukey post hoc test.

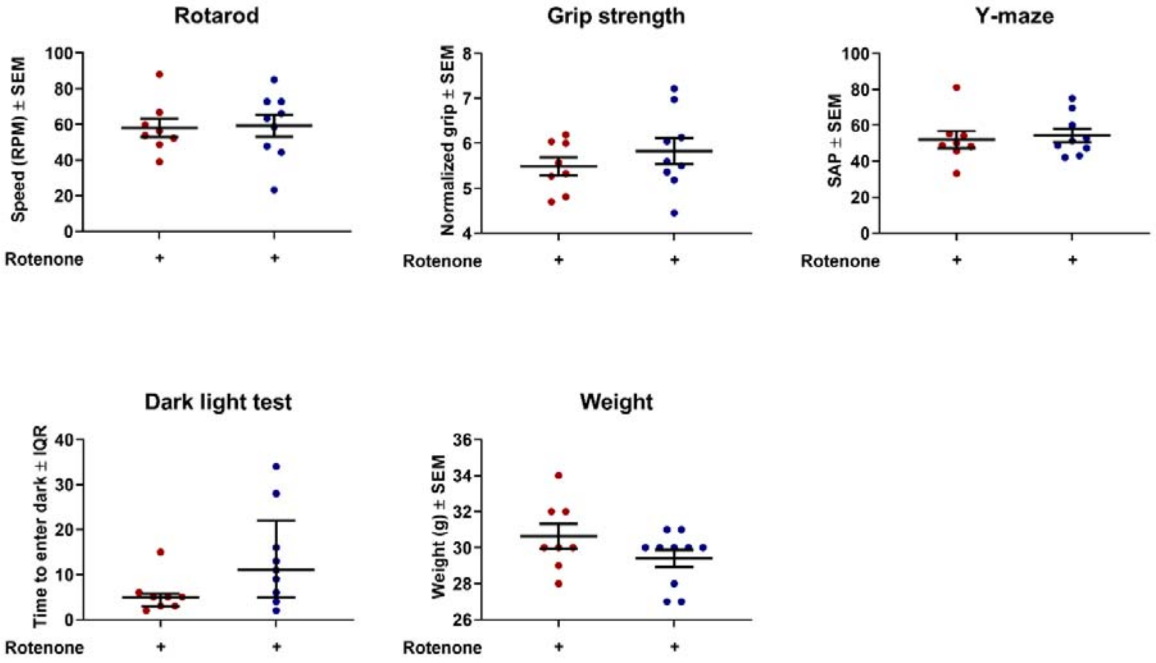

Supplementary Figure 3: Baseline following rotenone exposure for 32 days in the chronic rotenone wash-out study before treatment start

Wt mice were exposed to rotenone by oral gavage and tested at day 32 before randomization into treatment groups (group 1 and group 2, n=8-9). No significant differences were observed indicating proper randomization. Wt: wild type. Statistics: Two-tailed unpaired t-test or Mann-Whitney U test.

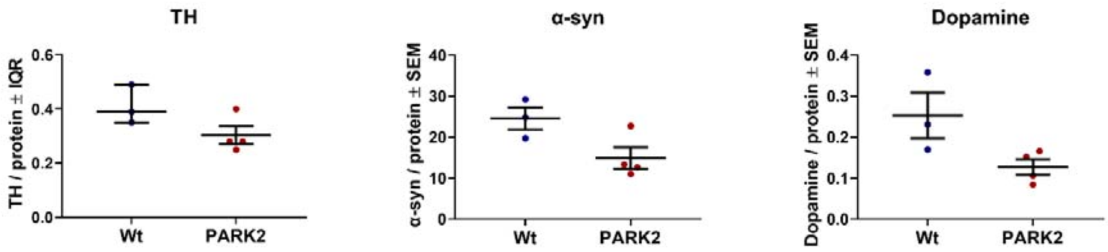

Supplementary Figure 4: PARK2 mice have no changes in TH, α-syn or dopamine protein concentrations compared to wild types.

The protein concentrations of TH, α-synuclein and dopamine in the midbrain were compared between Wt (n=3) and PARK2 mice (n=4) using ELISAs. No significant differences were detected. PARK2= PARK2 knockout mice, Wt=wild-type, RPM=rounds per minute, SAP=spontaneous alternation percentage. Statistics: Unpaired two-tailed t-test or Mann-Whitney U test.

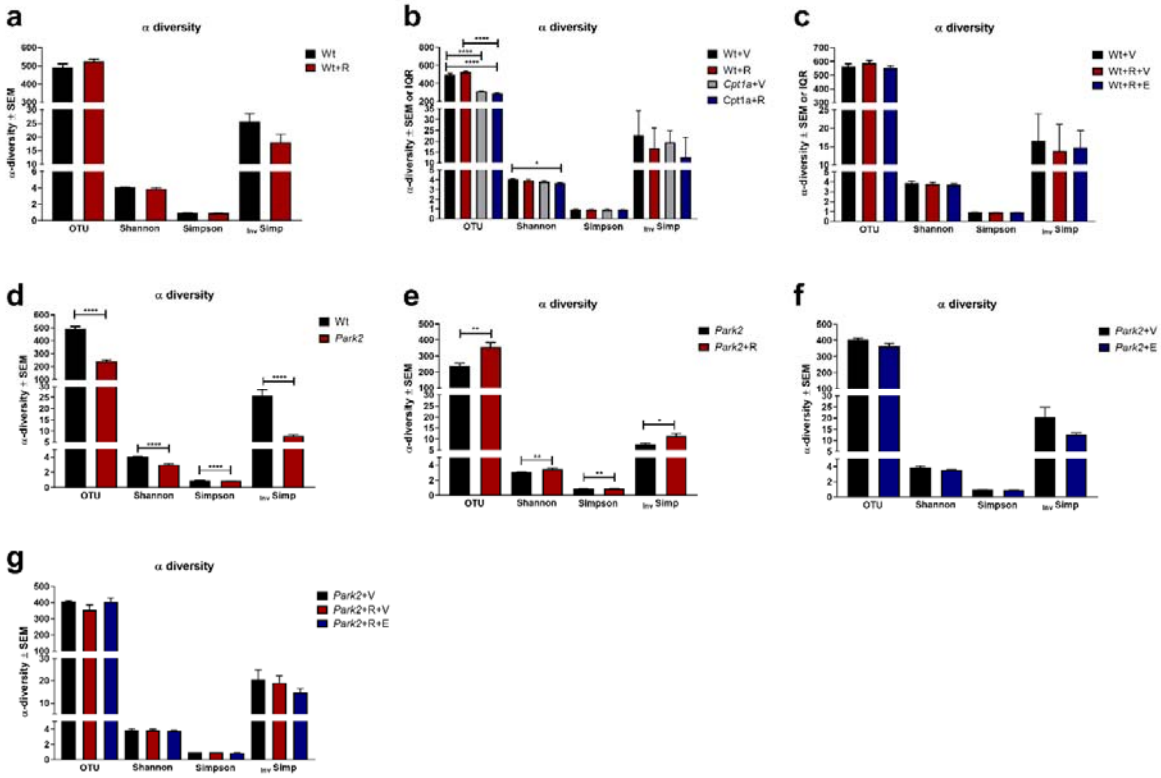

Supplementary Figure 5: Differences in α-diversity measures in P479L rotenone, Wt rotenone, PARK2, PARK2 with CPT1 inhibition and PARK2 rotenone experiments

a) α-diversity measures in Wt, and Wt+R mice at day 32

b) α-diversity measures in Wt, Wt+R, P479L+V (*Cpt1a*) and P479L+R (*Cpt1a*+R) mice at day 32

c) α-diversity measures in Wt+V, Wt+R+V, and Wt+R+E mice at day 62

d) α-diversity measures in Wt and PARK2 knockout mice (*Park2*)

e) α-diversity measures in PARK2+V (*Park2*+V) and PARK2+E (*Park2*+E) at day 22

f) α-diversity measures in PARK2+V (*Park2*), and PARK2+R mice (*Park2*+R) at day 32

f) α-diversity measures in PARK2+V (*Park2*+V), PARK2+R+V (*Park2*+R+V), and PARK2+R+E (*Park2*+R+E) mice at day 54

α-diversity measures in fecal gut microbiota samples from the different groups, presented in Figure 1-7. N=4-6, error bars represent the standard error of the mean (SEM). Data are representative of one experiment. Significant differences; *$p \leq 0.05$; **$p \leq 0.01$; ***$p \leq 0.001$; ****$p \leq 0.0001$. Wt=C57bl/6J mice, Cpt1a=*Cpt1a* P479L genotype, *Park2*=PARK2 knockout genotype, R=rotenone, V=Vehicle, E=etomoxir, OUT=operational taxonomic unit, Shannon=Shannon index, Inv^{Simp}=Inverse Simpson index. Statistics: Two-tailed unpaired t-test, two-way ANOVA followed by Tukey post hoc test, one-way ANOVA followed by Tukey post hoc or Kruskal-Wallis test followed by Dunns test.

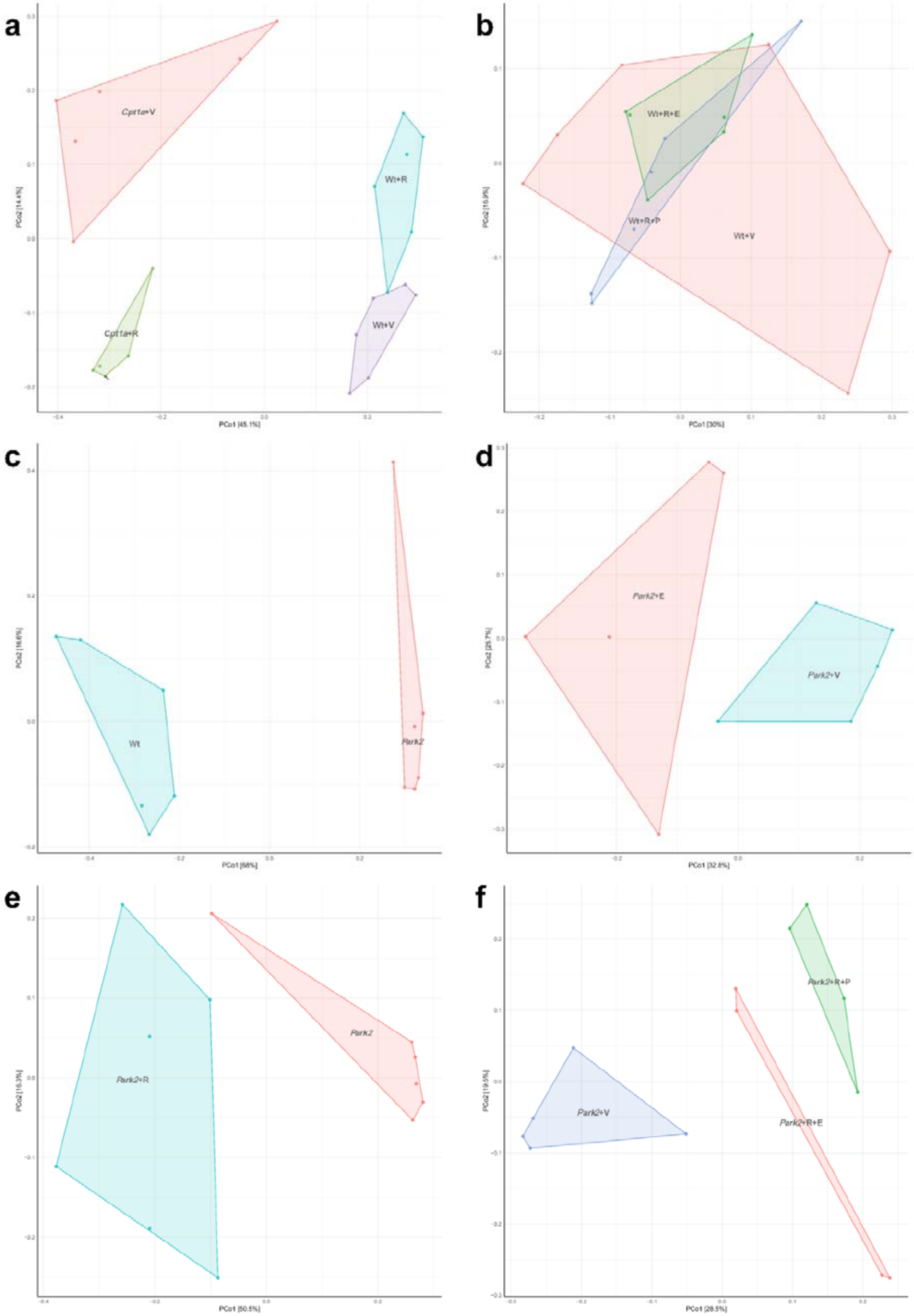
a
Cpt1a+V
Wt+R
Cpt1a+R
Wt+V
PCo1 [45.1%]
PCo2 [14.4%]
b
Wt+R+E
Wt+R+P
Wt+V
PCo1 [30%]
PCo2 [15.9%]
c
Wt
Park2
PCo1 [68%]
PCo2 [16.6%]
d
Park2+E
Park2+V
PCo1 [32.8%]
PCo2 [25.7%]
e
Park2+R
Park2
PCo1 [50.5%]
PCo2 [16.3%]
f
Park2+R+P
Park2+V
Park2+R+E
PCo1 [26.5%]
PCo2 [19.5%]

Supplementary Figure 6: Differences in β-diversity measures in P479L rotenone, Wt rotenone, PARK2, PARK2 with CPT1 inhibition and PARK2 rotenone experiments

a) β-diversity measures in Wt+V, Wt+R, P479L+V (*Cpt1a*) and P479L+R (*Cpt1a*+R) groups at day 32. **b)** β-diversity measures in Wt+V, Wt+R+V and Wt+R+E groups at day 62. **c)** β -diversity measures in Wt and PARK2 (*Park2*) mice. **d)** β -diversity measures in PARK2+V (*Park2*+V) and PARK2+E (*Park2*+E) mice at day 22. **e)** β-diversity measures in PARK2 (*Park2*) and PARK2+R (*Park2*+R) mice following 32 days of rotenone exposure. **f)** β-diversity measures in PARK2+V (*Park2*+V), PARK2+R+V(*Park2*+R+V) and PARK2+R+E (*Park2*+R+E) gice at day 54. β-diversity measures in fecal gut microbiome samples illustrated by principal coordinate analysis from the different groups presented in Supplementary Figure 5. The data illustrates intergroup differences indicating differences in the gut microbiome. N=4-6. Data are representative of one experiment. Wt=C57bl/6J mice, *Park2*=PARK2 knockout genotype, R=rotenone, V=Vehicle, E=etomoxir.

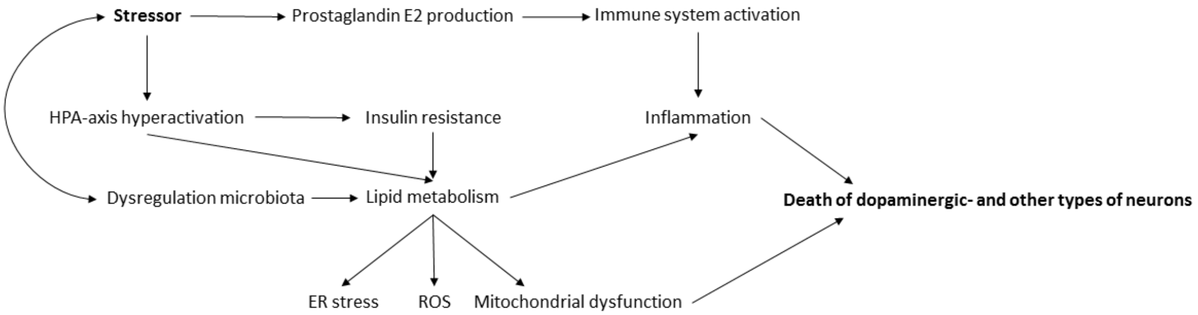

Supplementary Figure 7: Lipid metabolisms role in Parkinson's disease Stressors (e.g. micro bleeding, infection, hypoxia, depression) in genetic susceptible people results in a hyperactivation of the HPA-axis leading to production of glucocorticoids. The high level of glucocorticoids leads to insulin resistance shifting the metabolism towards lipids. Changes in the microbiota due to diet and genetics also facilitates lipid metabolism. Further, the stressors promote the production of prostaglandins leading to recruitment and activation of the immune system. The upregulated lipid metabolism stimulates the immune cells, further exacerbating inflammation, and results in endoplasmic reticulum stress, production of reactive oxygen species and mitochondrial dysfunction. All these processes lead to the development of PD.

Supplementary Table 1: Mean relative abundance at phyla, class, order, family and genus level.

Heatmaps illustrating the 6 most relative abundant phyla, 10 most relative abundance class, order and families and the 25 most abundant genus taxa. Mean relative abundance was calculated for each group based on n = 5-6 samples per group.

Phylum	WT+V	WT+R	P479L+V	P479L+R
Bacteroidetes	50,19	49,65	38,10	45,77
Firmicutes	39,16	42,49	55,09	32,11
Verrucomicrobia	5,62	0,08	2,90	19,02
Proteobacteria	4,21	4,94	0,79	0,32

	WT+V	WT+R	P479L+V	P479L+R
Actinobacteria	0,08	0,12	2,96	2,63
Cyanobacteria	0,60	2,53	0,14	0,11

**Class**	WT+V	WT+R	P479L+V	P479L+R
Bacteroidia	50,18781777	49,648433	38,1036551	45,7742303
Clostridia	21,60523404	25,5034331	37,0382763	24,7270679
Erysipelotrichia	9,488295421	12,1794415	12,4298517	5,24681341
Verrucomicrobiae	5,617373588	0,07741512	2,89869827	19,0214149
Bacilli	8,063498229	4,80676648	5,61774953	2,14054966
Coriobacteriia	0,080592381	0,12174277	2,96337694	2,6177806
Gammaproteobacteria	1,654465492	2,48745715	0,43732923	0,18517383
Melainabacteria	0,602808757	2,53215063	0,03326146	0,04722153
Deltaproteobacteria	1,404763064	1,15904851	0,32420106	0,12145402
Alphaproteobacteria	1,150652893	1,29319519	0,02623475	0,01230697

**Order**	WT+V	WT+R	P479L+V	P479L +R
Bacteroidales	50,18781777	49,648433	38,1036551	45,7742303
Clostridiales	21,60523404	25,5034331	37,0382763	24,7270679
Erysipelotrichales	9,488295421	12,1794415	12,4298517	5,24681341
Verrucomicrobiales	5,617373588	0,07741512	2,89839721	19,0214149
Lactobacillales	8,05462733	4,78404289	5,60969783	2,13098652
Coriobacteriales	0,080592381	0,12174277	2,96337694	2,6177806
Betaproteobacteriales	1,654465492	2,48633433	0,41715903	0,17263176
Gastranaerophilales	0,602808757	2,53215063	0,03326146	0,04722153
Desulfovibrionales	1,404763064	1,15904851	0,32420106	0,12145402
Rhodospirillales	1,150652893	1,29319519	0,00812721	0,00570399

**Family**	WT+V	WT+R	P479L+V	P479L+R
Muribaculaceae	30,07138121	34,5347922	22,5817636	29,6758998
Lachnospiraceae	15,59890584	20,3195876	33,6416962	22,126471
Erysipelotrichaceae	9,488295421	12,1794415	12,4298517	5,24681341
Prevotellaceae	7,136727066	4,95887531	9,32215508	8,91333764
Akkermansiaceae	5,617373588	0,07741512	2,89839721	19,0214149
Lactobacillaceae	8,05462733	4,76391557	5,42680401	2,00080067
Rikenellaceae	6,815802466	5,45556483	0,63796977	1,22771687
Bacteroidaceae	4,82562031	2,14037463	4,37657108	3,00929693
Ruminococcaceae	4,5289832	3,81306076	2,67413642	2,18650548
Marinifilaceae	1,061729103	2,22245591	0,86944194	2,26319925

**Genus**	WT+V	WT+R	P479L+V	P479L+R
bacterium	30,51343055	37,3873779	20,9024015	28,0053975
Lachnospiraceae NK4A136 group	9,166054191	10,4780734	6,40617363	0,39352286
Akkermansia	5,617373588	0,07741512	2,89839721	19,0214149
Dubosiella	9,100232622	10,7406615	1,6697498	0,00201258
Prevotellaceae UCG-001	4,953467924	3,45043818	7,93946952	6,19306487

Lactobacillus	8,05462733	4,76391557	5,42680401	2,00080067
Faecalibaculum	0,175578284	0,1492571	10,720254	5,23661893
Bacteroides	4,82562031	2,14037463	4,37657108	3,00929693
Alistipes	5,679980449	4,63239793	0,57884325	1,22771687
Alloprevotella	2,183259142	1,50843713	1,38268556	2,72027277
Odoribacter	1,061729103	2,22245591	0,86944194	2,26319925
f__Lachnospiraceae_OTU_35	0,043881397	0,12983813	2,77713463	3,40342933
Lachnospiraceae UCG-006	0,41557477	0,29345877	2,80631359	2,44630655
Muribaculum	1,465762402	0,8663899	1,70848297	1,72299732
f__Lachnospiraceae_OTU_32	0,124221861	0,05549183	2,41814881	3,36846102
Enterorhabdus	0,070738764	0,09440431	2,68538846	2,16675936
Parasutterella	1,654465492	2,48633433	0,38403531	0,14570015
f__Lachnospiraceae_OTU_19	0,001765958	0,00066332	2,32623469	2,38048425
f__Desulfovibrionaceae_OTU_25	1,388266459	1,13362215	0,259452	0,07743084
Lachnoclostridium	0,239883505	0,34534065	1,35274486	0,99235129
Ruminococcaceae UCG-014	1,34930675	1,39448721	0,00057926	0,00067207
o__Rhodospirillales_OTU_77	1,114319494	1,2659138	0,00812721	0,00570399
f__Lachnospiraceae_OTU_60	0,111983916	0,87184805	1,38120519	0
f__Lachnospiraceae_OTU_45	0,162422728	0,24849152	1,09703732	0,73947774
Roseburia	0,40461642	1,02163057	0,18875151	0,54966588

Supplementary Table 2: Mean relative abundance at phyla, class, order, family and genus level.

Heatmaps illustrating the 6 most relative abundant phyla, 10 most relative abundance class, order and families and the 25 most abundant genus taxa. Mean relative abundance was calculated for each group based on n = 6 samples per group.

**Phylum**	WT+V	WT+R+V	WT+R+E
Firmicutes	52,74	49,74	39,77
Bacteroidetes	32,13	34,73	36,07
Actinobacteria	10,11	12,15	14,03
Verrucomicrobia	3,94	2,03	8,98
Proteobacteria	0,62	0,97	0,78
Tenericutes	0,43	0,21	0,25

**Class**	WT+V	WT+R+V	WT+R+E
Bacteroidia	32,13	34,73	36,07
Clostridia	25,47	21,49	16,71
Bacilli	16,82	21,10	13,27
Actinobacteria	8,42	11,10	12,80
Erysipelotrichia	10,45	7,15	9,78
Verrucomicrobiae	3,94	2,03	8,98
Coriobacteriia	1,69	1,05	1,23
Gammaproteobacteria	0,42	0,64	0,63
Mollicutes	0,43	0,21	0,25
Deltaproteobacteria	0,19	0,31	0,14

**Order**	WT+V	WT+R+V	WT+R+E
Bacteroidales	32,13	34,73	36,07
Clostridiales	25,47	21,49	16,71
Lactobacillales	16,81	21,00	13,23
Bifidobacteriales	8,42	11,10	12,80
Erysipelotrichales	10,45	7,15	9,78
Verrucomicrobiales	3,94	2,03	8,98
Coriobacteriales	1,69	1,05	1,23
Betaproteobacteriales	0,42	0,63	0,63
Mollicutes RF39	0,43	0,21	0,25
Desulfovibrionales	0,19	0,31	0,14

**Family**	WT+V	WT+R+V	WT+R+E
Muribaculaceae	25,56	28,55	28,72
Lactobacillaceae	16,77	20,96	13,16
Lachnospiraceae	19,57	16,63	11,94
Bifidobacteriaceae	8,42	11,10	12,80
Erysipelotrichaceae	10,45	7,15	9,78
Akkermansiaceae	3,94	2,03	8,98
Ruminococcaceae	5,50	4,49	4,51
Prevotellaceae	4,56	3,82	5,07
Rikenellaceae	1,08	1,38	1,28
Eggerthellaceae	1,65	0,95	1,02

**Genus**	WT+V	WT+R+V	WT+R+E
bacterium	24,54	27,78	27,93
Lactobacillus	16,77	20,96	13,16
Bifidobacterium	8,42	11,10	12,80
Dubosiella	9,73	6,99	9,59
Lachnospiraceae NK4A136 group	6,40	7,68	5,81
Akkermansia	3,94	2,03	8,98
Prevotellaceae UCG-001	2,93	2,99	3,63
Ruminococcaceae UCG-014	2,89	1,93	2,97
[Eubacterium] xylanophilum group	2,03	1,58	1,00
Alloprevotella	1,63	0,83	1,44
Muribaculum	1,41	1,18	1,15
Alistipes	1,03	1,21	1,21
Enterorhabdus	1,32	0,68	0,77
f__Lachnospiraceae_OTU_89	1,16	0,85	0,43
Lachnospiraceae UCG-006	1,41	0,76	0,24
Lachnospiraceae UCG-001	1,00	0,73	0,32
Roseburia	0,67	0,56	0,60
Parasutterella	0,42	0,63	0,63
f__Lachnospiraceae_OTU_112	0,86	0,45	0,36

Odoribacter	0,52	0,60	0,48
GCA-900066575	0,51	0,54	0,38
Bacteroides	0,38	0,36	0,47
Lachnoclostridium	0,39	0,39	0,38
f__Lachnospiraceae_OTU_125	0,56	0,09	0,24
Parvibacter	0,33	0,27	0,24

Supplementary Table 3: Mean relative abundance at phyla, class, order, family and genus level.

Heatmaps illustrating the 6 most relative abundant phyla, 10 most relative abundance class, order and families and the 25 most abundant genus taxa. Mean relative abundance was calculated for each group based on n = 5-6 samples per group.

**Phylum**	Wt	*Park2*
Bacteroidetes	50,19	63,44
Firmicutes	39,16	18,03
Verrucomicrobia	5,62	17,54
Proteobacteria	4,21	0,77
Cyanobacteria	0,60	0,00
Actinobacteria	0,08	0,21

**Class**	Wt	*Park2*
Bacteroidia	50,19	63,44
Clostridia	21,61	15,24
Verrucomicrobiae	5,62	17,54
Erysipelotrichia	9,49	1,25
Bacilli	8,06	1,54
Alphaproteobacteria	1,15	0,77
Gammaproteobacteria	1,65	0,00
Deltaproteobacteria	1,40	0,00
Melainabacteria	0,60	0,00
Coriobacteriia	0,08	0,21

**Order**	Wt	*Park2*
Bacteroidales	50,19	63,44
Clostridiales	21,61	15,24
Verrucomicrobiales	5,62	17,54
Erysipelotrichales	9,49	1,25
Lactobacillales	8,05	1,45
Rhodospirillales	1,15	0,77
Betaproteobacteriales	1,65	0,00
Desulfovibrionales	1,40	0,00
Gastranaerophilales	0,60	0,00
Coriobacteriales	0,08	0,21

**Family**	Wt	*Park2*
Muribaculaceae	30,07	61,95
Lachnospiraceae	15,60	11,97
Akkermansiaceae	5,62	17,54
Erysipelotrichaceae	9,49	1,25
Lactobacillaceae	8,05	1,45
Rikenellaceae	6,82	1,48
Ruminococcaceae	4,53	2,64
Prevotellaceae	7,14	0,00
Bacteroidaceae	4,83	0,00
o__Rhodospirillales_OTU_77	1,11	0,77

**Genus**	Wt	*Park2*
bacterium	30,51	60,28
Akkermansia	5,62	17,54
Lachnospiraceae NK4A136 group	9,17	1,16
Lactobacillus	8,05	1,45
Dubosiella	9,10	0,01
Alistipes	5,68	1,48
Prevotellaceae UCG-001	4,95	0,00
Bacteroides	4,83	0,00
Muribaculum	1,47	2,04
Alloprevotella	2,18	0,00
f__Lachnospiraceae_OTU_19	0,00	1,95
o__Rhodospirillales_OTU_77	1,11	0,77
Ruminococcus 1	1,19	0,59
Parasutterella	1,65	0,00
Ruminococcaceae UCG-014	1,35	0,17
Faecalibaculum	0,18	1,23
f__Desulfovibrionaceae_OTU_25	1,39	0,00
Lachnospiraceae UCG-006	0,42	0,78
Rikenellaceae RC9 gut group	1,14	0,00
Odoribacter	1,06	0,00
f__Lachnospiraceae_OTU_67	0,02	1,01
Lachnoclostridium	0,24	0,58
[Eubacterium] xylanophilum group	0,62	0,07
f__Lachnospiraceae_OTU_1571	0,00	0,68
Ruminiclostridium 9	0,41	0,24

Supplementary Table 4: Mean relative abundance at phyla, class, order, and family and genus level.

Heatmaps illustrating the 6 most relative abundant phyla, 10 most relative abundance class, order and families and the 25 most abundant genus taxa. Mean relative abundance was calculated for each group based on n = 5 samples per group.

**Phylum**	*Park2*+V	*Park2*+E
Firmicutes	50,73224	43,5725
Bacteroidetes	43,09562	46,73289
Actinobacteria	4,366409	4,010336
Verrucomicrobia	0,658539	3,56525
Proteobacteria	1,088277	2,096884
Tenericutes	0,055437	0,017933

**Class**	*Park2*+V	*Park2*+E
Bacteroidia	43,09562	46,73289
Clostridia	30,89884	25,17046
Erysipelotrichia	12,68807	11,20903
Bacilli	6,041149	7,19061
Actinobacteria	4,134331	3,617753
Verrucomicrobiae	0,658539	3,56525
Gammaproteobacteria	1,087015	2,096105
p__Firmicutes_OTU_48	1,104181	0,002392
Coriobacteriia	0,232078	0,392583
Mollicutes	0,055437	0,017933

**Order**	*Park2*+V	*Park2*+E
Bacteroidales	43,09562	46,73289
Clostridiales	30,89884	25,17046
Erysipelotrichales	12,68807	11,20903
Lactobacillales	5,916134	7,175673
Bifidobacteriales	4,134331	3,617753
Verrucomicrobiales	0,658539	3,56525
Betaproteobacteriales	1,075327	2,085764
p__Firmicutes_OTU_48	1,104181	0,002392
Coriobacteriales	0,232078	0,392583
Bacillales	0,125015	0,014938

**Family**	*Park2*+V	*Park2*+E
Muribaculaceae	37,53282	44,80408
Lachnospiraceae	25,54973	20,84678
Erysipelotrichaceae	12,68807	11,20903
Lactobacillaceae	5,902387	7,160324
Ruminococcaceae	4,793301	3,515847
Bifidobacteriaceae	4,134331	3,617753

Bacteroidaceae	3,443165	1,570826
Akkermansiaceae	0,658539	3,56525
Burkholderiaceae	1,075327	2,085374
Rikenellaceae	2,116181	0,35758

**Genus**	*Park2*+V	*Park2*+E
bacterium	36,31336	43,83285
Dubosiella	12,23958	11,1474
Lachnospiraceae NK4A136 group	8,886118	4,859073
Lactobacillus	5,902007	7,160324
Bifidobacterium	4,134331	3,617753
Bacteroides	3,443165	1,570826
Lachnospiraceae UCG-001	2,638344	1,734084
Akkermansia	0,658539	3,56525
Parasutterella	1,075327	2,085374
Roseburia	1,646536	1,420936
Muribaculum	1,50159	1,408324
Lachnospiraceae UCG-006	0,551148	2,087316
Alistipes	2,116181	0,35758
Ruminiclostridium 6	1,414801	0,401487
[Eubacterium] xylanophilum group	1,2763	0,49996
f__Lachnospiraceae_OTU_109	0,434476	1,139723
Ruminococcaceae UCG-014	0,435115	0,672444
p__Firmicutes_OTU_48	1,104181	0,002392
Lachnoclostridium	0,451463	0,499582
f__Lachnospiraceae_OTU_35	0,566976	0,299572
GCA-900066575	0,500768	0,323972
Oscillibacter	0,472689	0,288284
f__Lachnospiraceae_OTU_145	0,617899	0,130345
f__Lachnospiraceae_OTU_97	0,133545	0,571013
Ruminiclostridium 9	0,345104	0,323258

Supplementary Table 5: Mean relative abundance at phyla, class, order, and family and genus level.

Heatmaps illustrating the 6 most relative abundant phyla, 10 most relative abundance class, order and families and the 25 most abundant genus taxa. Mean relative abundance was calculated for each group based on n = 5-6 samples per group.

**Phylum**	*Park2*	*Park2*+R
Bacteroidetes	63,44	46,54
Firmicutes	18,03	45,71
Verrucomicrobia	17,54	3,51
Proteobacteria	0,77	3,02

Cyanobacteria	0,00	0,85
Actinobacteria	0,21	0,23

**Class**	*Park2*	*Park2*+R
Bacteroidia	63,44	46,54
Clostridia	15,24	18,39
Verrucomicrobiae	17,54	3,51
Bacilli	1,54	14,16
Erysipelotrichia	1,25	13,16
Gammaproteobacteria	0,00	1,77
Alphaproteobacteria	0,77	0,75
Melainabacteria	0,00	0,85
Deltaproteobacteria	0,00	0,51
Coriobacteriia	0,21	0,18

**Order**	*Park2*	*Park2*+R
Bacteroidales	63,44	46,54
Clostridiales	15,24	18,39
Verrucomicrobiales	17,54	3,51
Lactobacillales	1,45	14,11
Erysipelotrichales	1,25	13,16
Betaproteobacteriales	0,00	1,76
Rhodospirillales	0,77	0,75
Gastranaerophilales	0,00	0,85
Desulfovibrionales	0,00	0,51
Coriobacteriales	0,21	0,18

**Family**	*Park2*	*Park2*+R
Muribaculaceae	61,95	43,67
Lachnospiraceae	11,97	14,29
Akkermansiaceae	17,54	3,51
Lactobacillaceae	1,45	14,09
Erysipelotrichaceae	1,25	13,16
Ruminococcaceae	2,64	3,16
Rikenellaceae	1,48	2,04
Burkholderiaceae	0,00	1,76
o__Rhodospirillales_OTU_77	0,77	0,74
bacterium	0,00	0,98

**Genus**	*Park2*	*Park2*+R
bacterium	60,28	43,71
Akkermansia	17,54	3,51
Lactobacillus	1,45	14,09
Faecalibaculum	1,23	11,45
Lachnospiraceae NK4A136 group	1,16	4,11

Muribaculum	2,04	1,55
Alistipes	1,48	2,04
Lachnospiraceae UCG-006	0,78	1,59
f__Lachnospiraceae_OTU_19	1,95	0,26
Parasutterella	0,00	1,76
Dubosiella	0,01	1,59
o__Rhodospirillales_OTU_77	0,77	0,74
Lachnospiraceae UCG-001	0,18	1,21
f__Lachnospiraceae_OTU_67	1,01	0,10
Ruminococcus 1	0,59	0,42
Ruminococcaceae UCG-014	0,17	0,82
Prevotellaceae UCG-001	0,00	0,82
f__Lachnospiraceae_OTU_1571	0,68	0,08
f__Lachnospiraceae_OTU_45	0,37	0,32
Lachnoclostridium	0,58	0,12
[Eubacterium] coprostanoligenes group	0,28	0,33
f__Lachnospiraceae_OTU_97	0,12	0,48
Marvinbryantia	0,20	0,39
Roseburia	0,18	0,40
Ruminiclostridium 9	0,24	0,33

Supplementary Table 6: Mean relative abundance at phyla, class, order, and family and genus level.

Heatmaps illustrating the 6 most relative abundant phyla, 10 most relative abundance class, order and families and the 25 most abundant genus taxa. Mean relative abundance was calculated for each group based on n = 4-6 samples per group.

**Phylum**	*Park2*+V	*Park2*+R+P	*Park2*+R+E
Firmicutes	50,73224	45,543485	49,8045147
Bacteroidetes	43,09562	47,204704	46,0755167
Actinobacteria	4,366409	0,6105591	0,58952919
Proteobacteria	1,088277	2,4533519	1,92879625
Verrucomicrobia	0,658539	2,8417634	0,93073978
Cyanobacteria	0,00038	1,0554301	0,20078188

**Class**	*Park2*+V	*Park2*+R+P	*Park2*+R+E
Bacteroidia	43,09562	47,204704	46,0755167
Clostridia	30,89884	32,351736	23,9701243
Erysipelotrichia	12,68807	2,6886506	14,7128619
Bacilli	6,041149	10,503098	11,1215286
Actinobacteria	4,134331	0,3447595	0,42012002
Verrucomicrobiae	0,658539	2,8417634	0,93073978
Gammaproteobacteria	1,087015	1,0650059	1,02833193
p__Firmicutes_OTU_48	1,104181	0	0

Alphaproteobacteria	0,000761	0,7451456	0,56814201
Melainabacteria	0,00038	1,0554301	0,20078188

**Order**	*Park2*+V	*Park2*+R+P	*Park2*+R+E
Bacteroidales	43,09562	47,204704	46,0755167
Clostridiales	30,89884	32,351736	23,9701243
Erysipelotrichales	12,68807	2,6886506	14,7128619
Lactobacillales	5,916134	10,500839	11,1197208
Bifidobacteriales	4,134331	0,3447595	0,42012002
Verrucomicrobiales	0,658539	2,8417634	0,93073978
Betaproteobacteriales	1,075327	1,0475236	1,02396705
p__Firmicutes_OTU_48	1,104181	0	0
Rhodospirillales	0	0,7451456	0,56621081
Gastranaerophilales	0,00038	1,0554301	0,20078188

**Family**	*Park2*+V	*Park2*+R+P	*Park2*+R+E
Muribaculaceae	37,53282	42,858392	39,7643094
Lachnospiraceae	25,54973	25,759615	18,3355813
Erysipelotrichaceae	12,68807	2,6886506	14,7128619
Lactobacillaceae	5,902387	10,472184	11,1044892
Ruminococcaceae	4,793301	5,0662807	4,62859526
Rikenellaceae	2,116181	2,5735309	4,7545928
Bacteroidaceae	3,443165	1,7722627	0,00065153
Bifidobacteriaceae	4,134331	0,3447595	0,42012002
Akkermansiaceae	0,658539	2,8417634	0,93073978
Burkholderiaceae	1,075327	1,0475236	1,02396705

**Genus**	*Park2*+V	*Park2*+R+P	*Park2*+R+E
bacterium	36,31336	43,867917	38,8601549
Lachnospiraceae NK4A136 group	8,886118	11,5331	7,32906061
Lactobacillus	5,902007	10,472184	11,1044892
Dubosiella	12,23958	2,3911373	5,08405999
Faecalibaculum	0,331563	0,2181526	9,47914561
Alistipes	2,116181	2,5735309	4,7545928
Bacteroides	3,443165	1,7722627	0,00065153
Bifidobacterium	4,134331	0,3447595	0,42012002
Muribaculum	1,50159	1,2783226	2,14899845
Lachnospiraceae UCG-001	2,638344	0,099219	1,35138557
Akkermansia	0,658539	2,8417634	0,93073978
Roseburia	1,646536	0,6675392	1,00963109
Parasutterella	1,075327	1,0475236	1,02267958
Lachnospiraceae UCG-006	0,551148	1,8407301	0,678197
[Eubacterium] xylanophilum group	1,2763	0,8152296	0,73206073
Ruminococcus 1	0,263462	0,7832138	1,23155422
Ruminococcaceae UCG-014	0,435115	0,2842636	1,03675622

f__Lachnospiraceae_OTU_45	0,351899	0,9701873	0,41725932
Ruminiclostridium 6	1,414801	0,0094367	0,01347151
Prevotellaceae UCG-001	0,001465	0	1,55531919
GCA-900066575	0,500768	0,3997807	0,50743233
A2	0,483577	0,1513586	0,64988675
p__Firmicutes_OTU_48	1,104181	0	0
Ruminiclostridium 9	0,345104	0,530339	0,41102364
f__Lachnospiraceae_OTU_35	0,566976	0,4239123	0,20230418
