# Supplementary material for: Inhibition of carnitine palmitoyl-transferase 1 is a potential target in a mouse model of Parkinson’s disease
Source: NPJ Parkinsons Dis. 2023 Jan 21;9:6. doi: 10.1038/s41531-023-00450-y (PMC9867753; doi:10.1038/s41531-023-00450-y)
Supplement: Supplementary file 2 — Reporting Summary [file 41531_2023_450_MOESM2_ESM.pdf]

nature portfolio

Corresponding author(s): John Dirk Vestergaard Nieland

Last updated by author(s): Nov 15, 2022

Reporting Summary

Nature Portfolio wishes to improve the reproducibility of the work that we publish. This form provides structure for consistency and transparency in reporting. For further information on Nature Portfolio policies, see our Editorial Policies and the Editorial Policy Checklist.

Statistics

For all statistical analyses, confirm that the following items are present in the figure legend, table legend, main text, or Methods section.

n/a	Confirmed	
☐	☒	The exact sample size (*n*) for each experimental group/condition, given as a discrete number and unit of measurement
☐	☒	A statement on whether measurements were taken from distinct samples or whether the same sample was measured repeatedly
☐	☒	The statistical test(s) used AND whether they are one- or two-sided<br>*Only common tests should be described solely by name; describe more complex techniques in the Methods section.*
☐	☒	A description of all covariates tested
☐	☒	A description of any assumptions or corrections, such as tests of normality and adjustment for multiple comparisons
☐	☒	A full description of the statistical parameters including central tendency (e.g. means) or other basic estimates (e.g. regression coefficient) AND variation (e.g. standard deviation) or associated estimates of uncertainty (e.g. confidence intervals)
☐	☒	For null hypothesis testing, the test statistic (e.g. *F*, *t*, *r*) with confidence intervals, effect sizes, degrees of freedom and *P* value noted<br>*Give P values as exact values whenever suitable.*
☒	☐	For Bayesian analysis, information on the choice of priors and Markov chain Monte Carlo settings
☒	☐	For hierarchical and complex designs, identification of the appropriate level for tests and full reporting of outcomes
☒	☐	Estimates of effect sizes (e.g. Cohen's *d*, Pearson's *r*), indicating how they were calculated

Our web collection on statistics for biologists contains articles on many of the points above.

Software and code

Policy information about availability of computer code

Data collection	No software was used except for the microbiome study. This is explained in the method section and in Trabjerg, M. S. et al. Dysregulation of metabolic pathways by carnitine palmitoyl-transferase 1 plays a key role in central nervous system disorders: experimental evidence based on animal models. Sci Rep 10, 1–19 (2020).
Data analysis	All data were analyzed using GraphPad Prism 8.0 software. LefSE is explained in Segata, N. et al. Metagenomic biomarker discovery and explanation. Genome Biol. 12, (2011).

For manuscripts utilizing custom algorithms or software that are central to the research but not yet described in published literature, software must be made available to editors and reviewers. We strongly encourage code deposition in a community repository (e.g. GitHub). See the Nature Portfolio guidelines for submitting code & software for further information.

Data

Policy information about availability of data

All manuscripts must include a data availability statement. This statement should provide the following information, where applicable:

- Accession codes, unique identifiers, or web links for publicly available datasets
- A description of any restrictions on data availability
- For clinical datasets or third party data, please ensure that the statement adheres to our policy

Data is available upon request to the corresponding author.

Human research participants

Policy information about studies involving human research participants and Sex and Gender in Research.

Reporting on sex and gender	*Use the terms sex (biological attribute) and gender (shaped by social and cultural circumstances) carefully in order to avoid confusing both terms. Indicate if findings apply to only one sex or gender; describe whether sex and gender were considered in study design whether sex and/or gender was determined based on self-reporting or assigned and methods used. Provide in the source data disaggregated sex and gender data where this information has been collected, and consent has been obtained for sharing of individual-level data; provide overall numbers in this Reporting Summary. Please state if this information has not been collected. Report sex- and gender-based analyses where performed, justify reasons for lack of sex- and gender-based analysis.*
Population characteristics	*Describe the covariate-relevant population characteristics of the human research participants (e.g. age, genotypic information, past and current diagnosis and treatment categories). If you filled out the behavioural & social sciences study design questions and have nothing to add here, write "See above."*
Recruitment	*Describe how participants were recruited. Outline any potential self-selection bias or other biases that may be present and how these are likely to impact results.*
Ethics oversight	*Identify the organization(s) that approved the study protocol.*

Note that full information on the approval of the study protocol must also be provided in the manuscript.

Field-specific reporting

Please select the one below that is the best fit for your research. If you are not sure, read the appropriate sections before making your selection.

☒ Life sciences ☐ Behavioural & social sciences ☐ Ecological, evolutionary & environmental sciences

For a reference copy of the document with all sections, see nature.com/documents/nr-reporting-summary-flat.pdf

Life sciences study design

All studies must disclose on these points even when the disclosure is negative.

Sample size	Samples sizes were based on similar studies in the literature (Zhou, Q. et al. Sulforaphane protects against rotenone-induced neurotoxicity in vivo: Involvement of the mTOR, Nrf2, and autophagy pathways. Sci. Rep. 6, 32206 (2016)., Yang, X., Qian, Y., Xu, S., Song, Y. & Xiao, Q. Longitudinal analysis of fecal microbiome and pathologic processes in a rotenone induced mice model of Parkinson's disease. Front. Aging Neurosci. 9, 1–12 (2018).), and were estimated using Gpower.
Data exclusions	Outliers were evaluated using Grubbs test, and if present, removed. Further, data from behaviour experiments were excluded if the animals did not apply to the respective protocols. This is explained in the method section.
Replication	P479L+R animal experiments were repeated twice and produced similar results. All other experiments were performed once.
Randomization	Animals were randomized before initiation of treatment. This is further explained in the method section.
Blinding	Investigators and video raters were blinded to genotype and treatment.

Reporting for specific materials, systems and methods

We require information from authors about some types of materials, experimental systems and methods used in many studies. Here, indicate whether each material, system or method listed is relevant to your study. If you are not sure if a list item applies to your research, read the appropriate section before selecting a response.

Materials & experimental systems

n/a	Involved in the study
☒	☐ Antibodies
☒	☐ Eukaryotic cell lines
☒	☐ Palaeontology and archaeology
☐	☒ Animals and other organisms
☒	☐ Clinical data
☒	☐ Dual use research of concern

Methods

n/a	Involved in the study
☒	☐ ChIP-seq
☒	☐ Flow cytometry
☒	☐ MRI-based neuroimaging

Animals and other research organisms

Policy information about studies involving animals; ARRIVE guidelines recommended for reporting animal research, and Sex and Gender in Research

Laboratory animals	C57BL/6J mice were obtained from Janvier Labs (Le Genest-Saint-Isle, France). Mice were acclimated for three weeks at 21 °C in a high barrier animal facility. Male B6.129S4-Prkntm1Shn/J 40 (JAX stock #006582) (Park2) mice were purchased from Jackson Laboratory (Bar Harbor, Maine, USA). The Park2 colony was maintained by breeding homozygote Park2 female together with Park2 male mice at the animal facility (Aalborg, Denmark). Only Park2 males were used for subsequent experiments. Mice were housed in groups of up to five in individually ventilated cages with a 12-hour light-dark cycle, and ad libitum access to food and water. Cpt1a P479L (Cpt1a) mice were generated in collaboration with Netherlands Cancer Institute (Mouse Clinic for Cancer and Aging transgenic facility, Amsterdam) as previously published 28. The colony was maintained at Aalborg University by breeding homozygote males, and females together. Only males were used for subsequent experiments.
Wild animals	*Provide details on animals observed in or captured in the field; report species and age where possible. Describe how animals were caught and transported and what happened to captive animals after the study (if killed, explain why and describe method; if released, say where and when) OR state that the study did not involve wild animals.*
Reporting on sex	Only males were used do to previous studies using the chronic rotenone model. This is further explained in the discussion and method.
Field-collected samples	*For laboratory work with field-collected samples, describe all relevant parameters such as housing, maintenance, temperature, photoperiod and end-of-experiment protocol OR state that the study did not involve samples collected from the field.*
Ethics oversight	All animal experiments within this study were based on the ARRIVE guidelines. The experimental procedures were approved by The Danish Animal Experiment Inspectorate (2017-15-0201-01328).

Note that full information on the approval of the study protocol must also be provided in the manuscript.
